# Temporal Decoupling: A New Paradigm of 4D Photo‐Responsive Carbon Dots Films With Modular Regulation of Color and Deformation

**DOI:** 10.1002/advs.76955

**Published:** 2026-08-06

**Authors:** Jianye Zhang, Meichen Meng, Zhimeng Ma, Mingbo Yue, Xiaoyu Xu, Chang Liu, Qiang Fu

**Affiliations:** ^1^ College of Engineering Qufu Normal University Rizhao Shandong China; ^2^ School of Chemistry and Chemical Engineering Qufu Normal University Qufu Shandong China; ^3^ College of Chemistry and Material Science Shandong Agricultural University Taian Shandong China

**Keywords:** carbon Dots, information encryption, photo‐responsive multicolor fluorescence, photothermally driven photoinduced deformation

## Abstract

To address the key bottlenecks of traditional photo‐responsive 4D materials, namely the inability to achieve independent regulation of photoinduced deformation and multicolor fluorescence, and the insufficient security of encryption systems, this study takes naphthalimide‐functionalized carbon dots (CDs) as the core functional unit, and for the first time proposes and verifies a novel energy regulation strategy using the long‐lived S_1_ transition as a “kinetic capacitor”. This strategy enables dual‐channel programmable shunting of excited‐state energy: radiative relaxation for fluorescence emission, and non‐radiative relaxation‐generated photothermal effect for deformation driving. By adjusting the feeding ratio of triethanolamine to tune the HOMO‐LUMO energy gap, multicolor fluorescence from blue to orange‐red is achieved. On this basis, this study successfully constructs two independent regulation systems, including photothermally driven deformation and S_1_ state energy level‐tuned photo‐responsive multicolor fluorescence, and fabricates 4D photo‐responsive smart films with the characteristic of temporally decoupled integration of photo‐responsive multicolor fluorescence and photoinduced deformation. Relying on the irreversible photo‐response properties and the difference in photo‐response time of the materials, a three‐dimensional dynamic single‐use anti‐counterfeiting model is established, which provides a new approach for the modular design of 4D smart materials and their high‐end anti‐counterfeiting applications.

## Graphical abstract

This work demonstrates a novel energy regulation strategy using the long‐lived S₁ state as a “kinetic capacitor” to achieve temporally decoupled integration of photoinduced deformation and multicolor fluorescence in naphthalimide‐functionalized carbon dots. This dual‐channel programmable shunting of excited‐state energy establishes a dynamic single‐use anti‐counterfeiting model, opening new avenues for 4D smart material design.

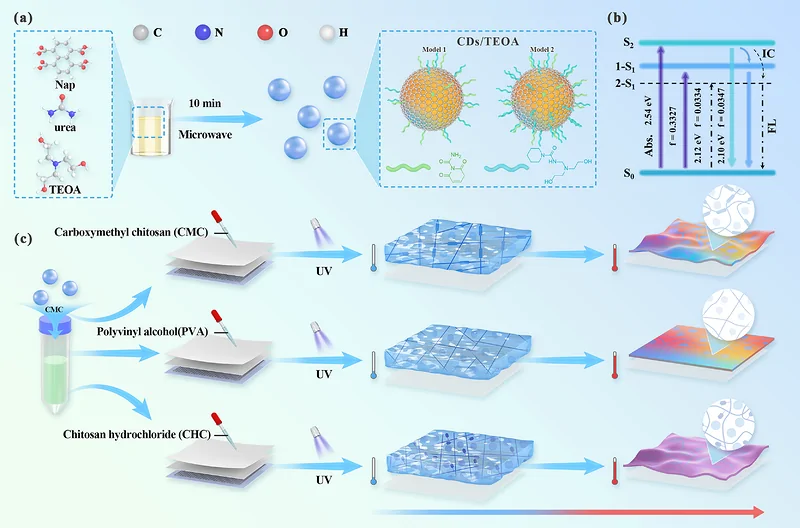

## 1 Introduction

4D smart materials are constructed by introducing time as the fourth dimension on the basis of 3D materials [1, 2, 3, 4]. They can realize the dynamic and programmable evolution of three‐dimensional structures and functions over time under external stimuli, and serve as the core support for cutting‐edge fields such as smart devices [5, 6, 7]. Among various stimulus‐response modes, light‐driven 4D materials have become a research hotspot owing to their advantages of non‐contact manipulation and high controllability [8, 9, 10, 11]. In particular, optomechanically coupled 4D materials with both photoinduced deformation and dynamic optical regulation enable the seamless integration of “optical signal input–time‐dependent optical output–dynamic evolution of three‐dimensional structure”, breaking through the functional boundaries of traditional single‐response materials. They have shown tremendous application potential in fields including dynamic information encryption [12, 13, 14, 15], soft robotics [16, 17, 18], and biomimetic intelligent systems [19, 20]. However, current 4D materials generally lack such integrated capability [21, 22]. Meanwhile, most existing encryption systems are based on reversible responses, which have core potential safety hazards of repeated decryption and illegal replication of information [23, 24, 25].

Current light‐driven 4D materials still face multiple core bottlenecks. In terms of performance regulation methods, the traditional regulation of photoinduced deformation mostly relies on mechanisms such as orientational rearrangement of liquid crystal elastomers, and these mechanisms suffer from inherent drawbacks including complex fabrication processes and slow response kinetics. The traditional regulation of photo‐responsive multicolor fluorescence is mostly based on energy level tuning of a single luminescent center, making it difficult to achieve wide‐color‐gamut time‐dependent fluorescence emission and leverage the key multi‐dimensional advantage of 4D responses [26, 27, 28]. More critically, existing fluorescence‐deformation dual‐response systems generally have severe functional coupling: the two properties are linked to the same molecule or structural unit, making independent regulation and decoupling unachievable [29, 30, 31]. For example, Yang et al. reported a spiropyran photochromic moiety‐based hydrogel system that enables synchronous multicolor fluorescence switching and bidirectional photoinduced deformation under a single light stimulus [32]. However, the fluorescence regulation and mechanical deformation of this system are completely coupled to the photoisomerization process of spiropyran molecules, and the system cannot achieve complete decoupling of the two functions. This makes it difficult to meet the core requirement for independent, multi‐dimensional performance manipulation of 4D smart materials.

Carbon dots (CDs), as a novel class of zero‐dimensional carbon‐based luminescent nanomaterials, possess core advantages including facile preparation, excellent optical properties and favorable biocompatibility, and have exhibited tremendous application potential in fields such as optoelectronic devices and information encryption [33, 34, 35, 36]. In recent years, researchers have achieved precise regulation of photo‐responsive fluorescence emission through strategies including precursor design, and constructed a series of dynamic fluorescence information encryption systems [37, 38]. Li et al. realized intrinsic photochromic luminescence in the range of 425–640 nm based on the surface‐state dual‐emission center mechanism [39]. However, existing studies are still focused on the single‐dimensional regulation of optical properties, and there are few reports on CDs‐based optomechanically coupled 4D materials. The 4D multi‐response integration of photoinduced deformation and multicolor fluorescence, as well as the independent decoupling regulation of the two properties, have not yet been realized [40, 41]. Meanwhile, most existing studies focus on radiative transitions with high oscillator strength, neglecting the energy regulation potential of excited states with weak transitions. They fail to resolve the energy competition between luminescence and deformation from the fundamental origin of the excited‐state electronic structure, which restricts the expansion of their applications in the field of 4D smart devices.

In the conventional understanding, “functional decoupling” generally refers to complete independence at the chemical structural level, meaning two functions are implemented by independent functional units and can be triggered by distinct stimuli respectively [42, 43]. However, in single‐field‐driven 4D smart material systems, such strict structural decoupling is both difficult to achieve and not a necessary prerequisite for the independent regulation of multiple responses. Building upon the core attribute of 4D materials that “time serves as the fourth dimension”, this work proposes a novel regulation paradigm of temporal decoupling: under single UV excitation, photoinduced chromism and deformation rely on two separate microscopic mechanisms and kinetic pathways with distinctly different response rates and time windows, thereby enabling staged presentation, programmable control and modular combination of material functions. There are essential differences between the present system and conventional coupled dual‐response systems. In conventional systems, both fluorescence chromism and photoinduced deformation originate from the same chemical reaction of a single functional molecule; they are tightly coupled and concomitant with synchronized response kinetics, and cannot be regulated independently [29, 30, 32]. In contrast, the present system only shares the initial excitation energy, while its response mechanisms, regulation pathways and kinetic behaviors are completely independent. They can be selectively optimized via different structural parameters, and independent switching of a single function can even be realized through rational matrix design. Benefiting from this temporal decoupling, the two photoresponsive signals are released sequentially under continuous UV irradiation, overcoming the critical flaw of conventional synchronous dual‐response materials where all encrypted information is fully exposed in a single illumination. The time gap between fluorescence evolution and macroscopic deformation enables staged hierarchical decryption: short irradiation only reveals superficial fluorescence coding, while deep hidden information is accessible only after prolonged irradiation‐induced film peeling, greatly raising the anti‐counterfeiting security threshold. Furthermore, this matrix‐mediated temporal decoupling strategy eliminates the need for multiple excitation sources and complex molecular design required for structural decoupling, simplifying film fabrication and providing a universal solution for information security scenarios including high‐end brand traceability and classified document encryption [44]. This strategy provides a new approach for the multi‐response modular regulation of 4D materials under a single excitation source.

Taking naphthalimide‐functionalized CDs (CDs/TEOA, triethanolamine, TEOA) as the core, this work, for the first time, leverages the energy regulation characteristics of the long‐lived S_1_ transition with low oscillator strength, and proposes a brand‐new design concept of using the S_1_ transition as a “kinetic capacitor”. This long‐lived S_1_ state can act as a buffer unit for excited‐state energy, realizing dual‐channel independent shunting of excited‐state energy: fluorescence emission is achieved via radiative relaxation, and the photothermal effect is generated through non‐radiative relaxation. This dual‐channel shunting strategy simultaneously meets the energy requirements for multicolor luminescence and photoinduced deformation. By tuning the feeding ratio of TEOA to regulate the S_1_ state energy level of the CDs, five types of photo‐responsive fluorescent CDs with emission colors ranging from orange‐red to green are obtained. Relying on the difference in the time scales of multicolor photoinduced chromism and photoinduced deformation, we fabricated 4D smart CDs/TEOA composite films featuring the property of temporal dimension decoupling. Meanwhile, relying on the irreversible photo‐responsive properties of the system, a single‐use encryption system with both a time‐dimension key and multi‐modal response is established. Starting from the origin of the excited‐state electronic structure, this work establishes a brand‐new methodology for performance decoupling of 4D smart materials, and provides a new approach for the modular design of multifunctional photo‐responsive materials and their high‐end smart anti‐counterfeiting applications.

## 2 Results and Discussion

### 2.1 CDs‐Based Composite Materials With Photo‐Responsive Fluorescence Chromism and Photoinduced Deformation

In this study, using 1,4,5,8‐naphthalenetetracarboxylic acid, urea, and TEOA as precursors, a series of CDs/TEOA‐1∼5 was rapidly synthesized via a microwave‐assisted method. On this basis, the as‐prepared CDs/TEOA series were compounded with polymer matrices to fabricate CDs‐based composite films with dual functions of photoinduced multicolor fluorescence chromism and photoinduced deformation. Under UV light irradiation, this series of materials can not only realize full‐spectrum gradient tuning of fluorescence color from blue to orange‐red, but also produce macroscopic bending deformation visible to the naked eye. More critically, there is an inherent time scale difference between the two response processes of fluorescence chromism and deformation, which successfully realizes the temporal decoupling of the two processes. The monolithic functional integration of “optical signal input—time‐dependent optical output—dynamic evolution of three‐dimensional structure” is completed, and the 4D dynamic integration of triple responses including photoinduced chromism, photo‐responsive multicolor fluorescence, and photoinduced deformation is achieved (Scheme 1). This work provides the core material foundation for high‐security dynamic information encryption applications.

**SCHEME 1:**
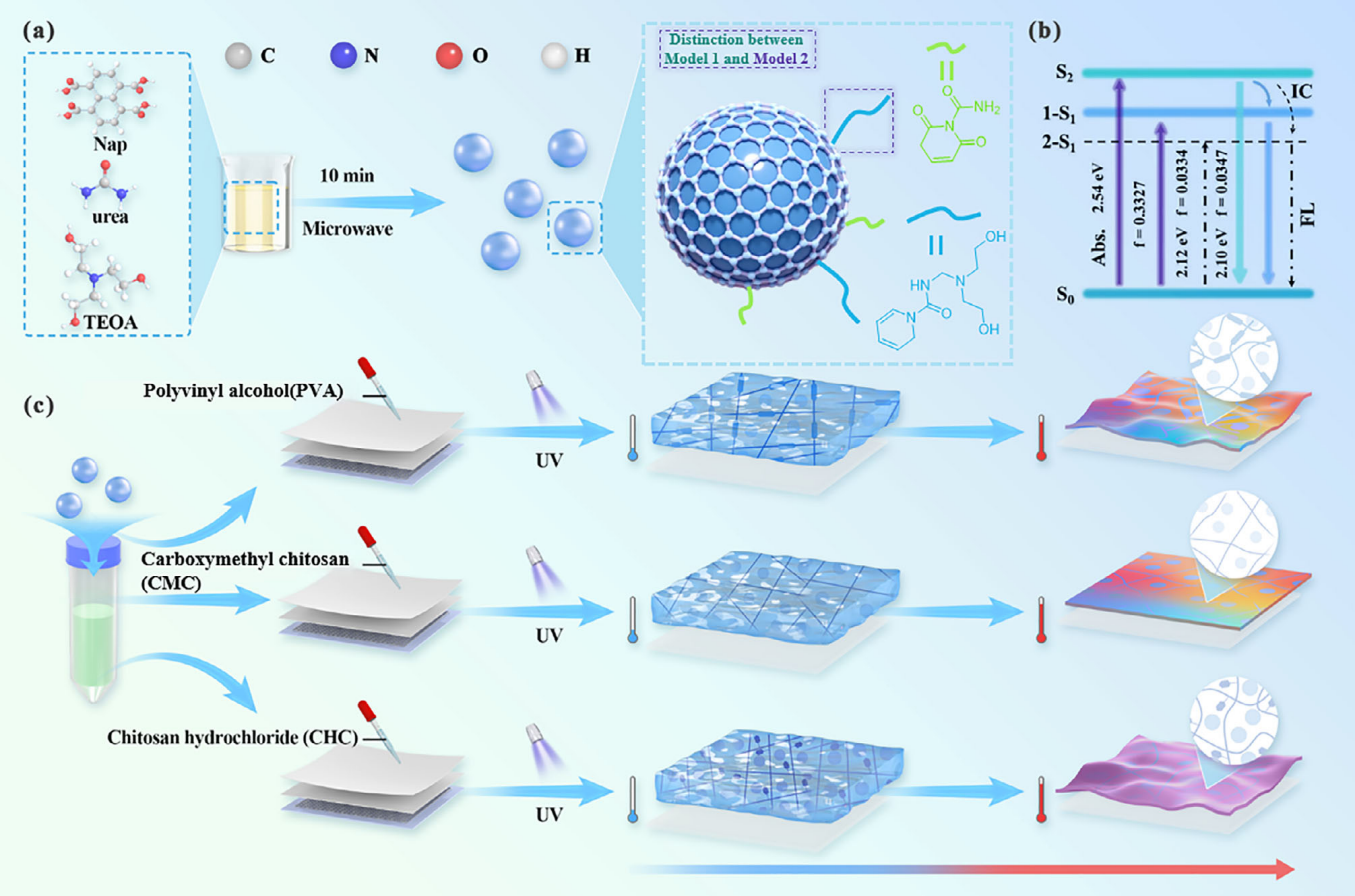
Schematic illustration of the preparation and photophysical mechanism of CDs/TEOA and its photo‐responsive composite films. (a) Microwave‐assisted synthesis process of CDs/TEOA; (b) Energy level transition and photophysical process of CDs/TEOA under UV irradiation; (c) Preparation of CDs/TEOA composite films and their response process of photoinduced deformation (photothermal effect) and photoinduced fluorescence chromism under UV irradiation.

### 2.2 Synthesis and Structure Characterization of CDs/TEOA

In this study, a series of CDs/TEOA samples were prepared via a microwave‐assisted method. By tuning the feeding ratio of TEOA, five groups of CDs/TEOA samples with distinct structural characteristics (CDs/TEOA‐1 to CDs/TEOA‐5) were obtained. To clarify the microstructure, crystalline features, functional group composition and elemental chemical states of the as‐prepared sample series, systematic structural characterizations were performed using transmission electron microscopy (TEM), X‐ray diffraction (XRD), Raman spectroscopy, Fourier transform infrared spectroscopy (FTIR) and X‐ray photoelectron spectroscopy (XPS). To investigate the morphology and microstructural characteristics of the as‐prepared samples, TEM morphological characterization was carried out for the five CDs/TEOA samples. The results are shown in Figure S1: CDs prepared with all feeding ratios exhibit a monodispersed quasi‐spherical morphology without obvious agglomeration, and feature a uniform particle size distribution. With the increase in the feeding volume of TEOA, the average particle size of the CDs shows a regular decreasing trend. The average particle sizes of CDs/TEOA‐1 to CDs/TEOA‐5 are 2.63, 2.61 2.56, 2.53 and 2.50 nm, respectively. The reduction in particle size indicates that the addition of TEOA can promote the carbonization of the carbon source and the growth of conjugated structures, leading to a gradual increase in the size of the sp^2^ carbon core and an enhanced degree of graphitization [45]. In the high‐resolution transmission electron microscopy (HRTEM) images in the insets, lattice fringes can be observed for all CDs samples, with a consistent lattice spacing of 0.21 nm. This value corresponds to the (100) crystal plane of graphite, confirming that all the CDs/TEOA samples in the series possess a highly crystalline graphitized structure [46, 47, 48, 49, 50].

The XRD characterization results are shown in Figure 1a. All samples exhibited a broad characteristic diffraction peak at 2θ ≈ 23°, a typical feature of the graphitized structure of carbon‐based nanomaterials, which is in good agreement with the HRTEM characterization results [51, 52]. In addition, the relatively sharp peaks at 28.15° and in the range of 10° to 22° can be attributed to the XRD peaks of molten urea [53, 54, 55]. Raman spectroscopy was employed to investigate the regulating effect of TEOA feeding amount on the graphitization degree and structural defects of the series of CDs/TEOA, with the results shown in Figure S2. CDs/TEOA‐1 to CDs/TEOA‐5 all exhibit two characteristic peaks at 1420 cm^−^^1^ and 1608 cm^−^^1^, which correspond to the disordered D band (sp3 hybridized carbon) and crystalline G band (sp2 hybridized carbon) of carbon materials, respectively [56]. The intensity ratio (ID/IG) of CDs/TEOA‐1 to CDs/TEOA‐5 decrease gradually, with values of 0.47, 0.45, 0.44, 0.40 and 0.350.35, 0.40, respectively, indicating that the increase of TEOA feeding ratio can effectively enhance the graphitization degree of CDs and reduce the defect sites in the carbon lattice. This trend is in good agreement with the typical graphitized structure observed by TEM [47].

**FIGURE 1:**
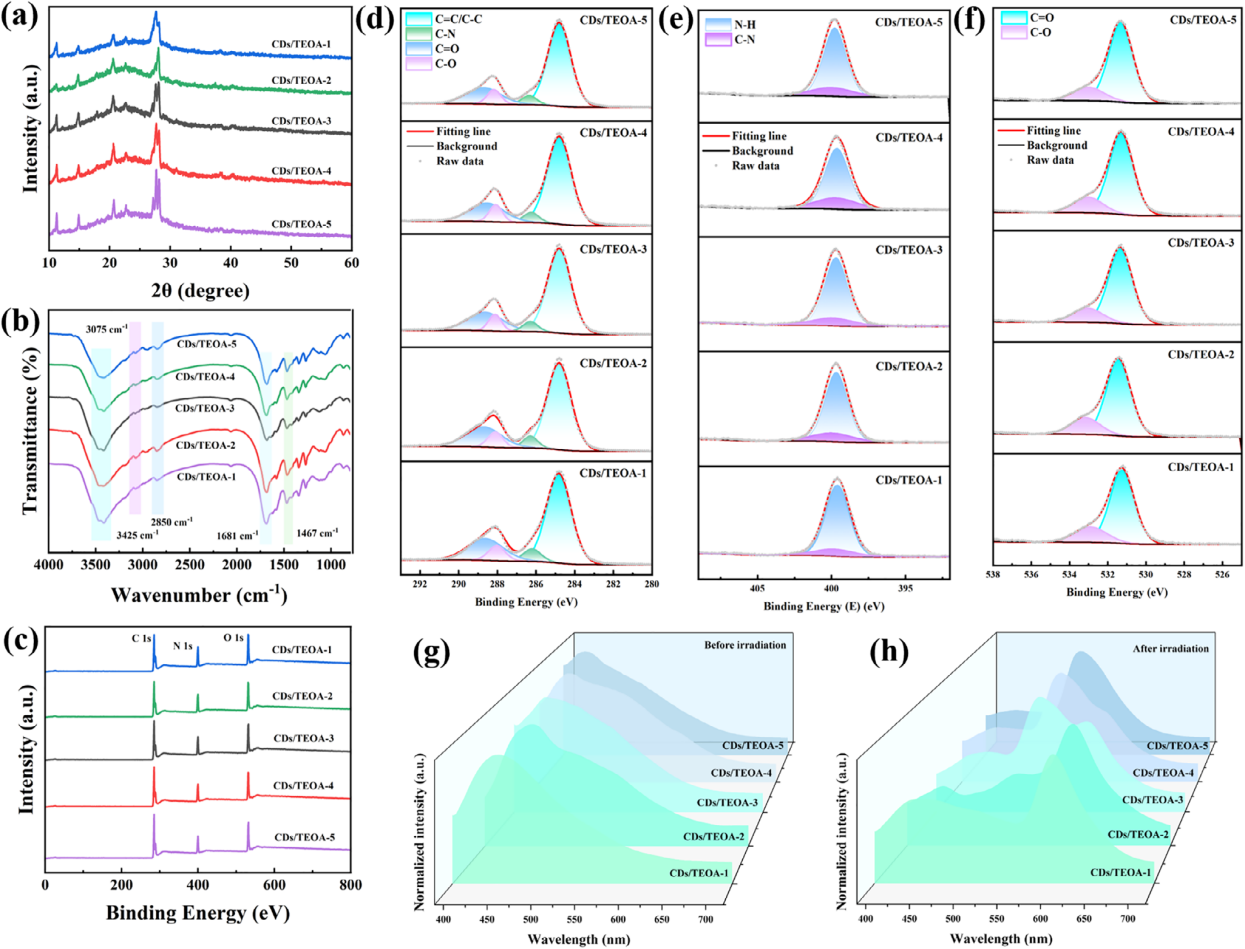
Characterization of the structure and optical properties of the CDs/TEOA‐1–5 series samples: (a) XRD patterns; (b) FTIR spectra; (c) XPS full survey spectra; (d) High‐resolution peak‐fitting XPS spectra of C 1s; (e) High‐resolution peak‐fitting XPS spectra of N 1s; (f) High‐resolution peak‐fitting XPS spectra of O 1s; (g) Normalized fluorescence emission spectra of the CDs/TEOA‐1–5 samples before UV light irradiation; (h) Normalized fluorescence emission spectra of the CDs/TEOA‐1–5 samples after UV light irradiation.

Systematic analysis of the surface functional groups and chemical bond structures of the five CDs/TEOA samples was performed via FTIR characterization, with the results shown in Figure 1b. The FTIR spectra of CDs/TEOA‐1 to CDs/TEOA‐5 exhibited highly consistent characteristic absorption peaks, indicating that the variation in TEOA dosage does not alter the main types of functional groups on the surface of the CDs/TEOA. Among them, the broad absorption peak at 3425 cm^−^^1^ is ascribed to the stretching vibrations of N─H and O─H groups [57], the weak absorption peak at 3075 cm^−^^1^ corresponds to the C─H stretching vibration of aromatic rings [58], and the characteristic peak at 2850 cm^−^^1^ originates from the symmetric stretching vibration of ‐CH_2_‐ [59], which confirms the successful modification of TEOA molecular chains on the surface of CDs/TEOA‐1 to 5. The two strong characteristic peaks at 1681 and 1467 cm^−^^1^ correspond to the characteristic stretching vibrations of the O═C─N bond and C─N bond, respectively [60]. Importantly, no obvious absorption signal was observed for all five CDs/TEOA samples in the range of 1740–1770 cm^−^^1^, which corresponds to the characteristic stretching vibration of the carboxyl group [61]. This indicates that the carboxyl groups in the precursors have fully participated in the cyclization reaction, and that the intact naphthalimide structure has been successfully constructed in all CDs/TEOA systems with different TEOA ratios, providing the core structural basis for their subsequent light‐responsive performance [62].

XPS was employed to characterize the surface elemental composition and chemical states of the five CDs/TEOA samples, with the results of the full survey spectra shown in Figure 1c. All samples exhibit three characteristic peaks near 284.8, ∼400, and ∼531 eV, which correspond to the characteristic orbitals of C 1s, N 1s and O 1s, respectively. The quantitative elemental analysis results show that the atomic proportion of C element in CDs/TEOA‐1 to CDs/TEOA‐5 presents an increasing trend, rising from 61.17% to 65.91%; while the atomic proportions of N and O elements decrease synchronously, with N content dropping from 18.80% to 15.21% and O content decreasing from 20.03% to 18.89% (Table S1). This variation trend is consistent with the molecular structural characteristics of TEOA, which further confirms the successful incorporation of TEOA. The fine chemical bond structure of the samples was further elucidated through peak fitting of the high‐resolution spectra of each element. The peak fitting results of the high‐resolution C 1s spectra are shown in Figure 1d and Table S2, and four characteristic peaks can be deconvoluted for all samples: the main peak at a binding energy of 284.8 eV is assigned to the C = C/C‐C bonds in the carbon skeleton, and its peak area proportion gradually increased from 63.74% for CDs/TEOA‐1 to 67.77% for CDs/TEOA‐5 with the decrease of TEOA feeding amount; the characteristic peak at 286.25–286.65 eV corresponds to the C─N bond, the peak at 288.09–288.18 eV is attributed to the C─O bond, and the peak at 288.56∼288.75 eV corresponds to the C═O bond. Among them, the peak area proportions of C─N and C─O bonds decreased synchronously with the reduction of TEOA feeding amount, which are mutually consistent with the quantitative analysis results of N and O elements from the XPS survey spectra, further confirming the regulation effect of TEOA dosage on the surface chemical composition of CDs/TEOA (Table S2). The peak fitting results of the high‐resolution N 1s spectra are shown in Figure 1e and Table S3. Two characteristic peaks are deconvoluted for all samples at 399.6–399.71 eV and 400.6–401.2 eV, which are ascribed to surface N─H bonds and skeletal C─N bonds of CDs/TEOA, respectively. The peak fitting results of the high‐resolution O 1s spectra are shown in Figure 1f. Two characteristic peaks can be deconvoluted for all five CDs/TEOA samples: the main peak at the binding energy of 531.24–531.33 eV is ascribed to the C═O double bond structure, and the secondary peak at 532.9–533.05 eV corresponds to the C─O single bond structure. Among them, the peak area proportion of C═O bonds increase with the decrease of TEOA feeding amount, rising from 78.53% for CDs/TEOA‐1 to 82.62% for CDs/TEOA‐5, while the proportion of C─O bonds decreases synchronously. This result is in high agreement with the peak fitting results of the C 1s spectra (Table S4).

### 2.3 Intrinsic Photo‐Responsive Performance Characterization of CDs/TEOA

To further investigate the optical properties of CDs/TEOA, aqueous solutions of CDs/TEOA with different ratios were prepared, and both their fluorescence and daylight color were found to change under continuous UV irradiation. The UV–vis absorption spectra of the samples before and after 365 nm UV irradiation were characterized (Figure S3). After irradiation, all samples exhibited distinct new absorption peaks in the visible region of 400–610 nm, which were presumed to originate from the generation of new species. Moreover, with the decrease in TEOA dosage, the UV irradiation time required for absorption peak saturation increased gradually, which were 70, 100, 160, 220, and 280 s for CDs/TEOA‐1 to CDs/TEOA‐5, respectively. With the enhancement of the absorption peaks, the solutions gradually changed from colorless and transparent to uniform yellowish‐brown under daylight (Figure S4).

The photoinduced fluorescence chromism of the five CDs/TEOA aqueous solutions was systematically characterized by steady‐state fluorescence emission spectroscopy. In the initial state without UV irradiation, all samples showed a blue fluorescence emission peak near 455 nm (Figure 1g). After UV irradiation, the intensity of the peak at 455 nm decreased continuously, while new peaks appeared at 535 and 605 nm. The relative intensities of the two new peaks changed regularly with the TEOA dosage (Figure 1h), corresponding to fluorescence colors of orange‐red, orange‐yellow, yellow, yellow‐green, and green in sequence (Figure S4). With the prolongation of UV irradiation time, the fluorescence spectra of CDs/TEOA‐1 and CDs/TEOA‐2 showed similar evolution trends: the intensity of the peak at 440 nm decreased continuously, while the intensity of the new peak at 605 nm increased gradually, and the latter eventually dominated, showing significant fluorescence red‐shift and color switching (Figure S5a,b). The CIE 1931 chromaticity diagram showed that their chromaticity coordinates shifted continuously from the blue region to the long‐wavelength direction, and finally appeared orange‐red and orange‐yellow (Figure S6a,b). CDs/TEOA‐3 and CDs/TEOA‐4 exhibited similar fluorescence evolution laws. With the extension of irradiation time, new peaks appeared at both 535 and 605 nm, and the peak at 535 nm eventually dominated, while the peak at 605 nm was a weak shoulder peak. In addition, the intensity of the 605 nm shoulder peak of CDs/TEOA‐4 was lower (Figure S5c,d), corresponding to yellow and yellow‐green fluorescence colors (Figure S6c,d). For CDs/TEOA‐5, with the prolongation of irradiation time, the peaks at 535 and 605 nm increased synchronously at first, then the peak at 605 nm gradually disappeared, and finally only the green peak at 535 nm dominated absolutely (Figure S5e), with the fluorescence color gradually turning green (Figure S6e).

### 2.4 Photo‐Responsive Performance and Mechanism of CDs/TEOA@PVA Composite Films

To investigate the photo‐responsive behavior of CDs/TEOA in polymer matrices, five CDs/TEOA samples were first blended with polyvinyl alcohol (PVA) solution to prepare five CDs/TEOA@PVA composite films. The chemical structure and composite mechanism of the composite films were characterized by FTIR spectroscopy. As shown in Figure 2a, the five CDs/TEOA@PVA composite films exhibited basically consistent structural variation trends. By comparing the FTIR spectra of pure CDs/TEOA, pure PVA and composite films, no new characteristic absorption peaks are observed in the composite films, demonstrating that the intrinsic chemical structure of CDs/TEOA is not damaged during the composite preparation process. All composite films completely retained the characteristic stretching vibration peaks of O═C─N and C─N bonds corresponding to the naphthalimide ring, which provided a core structural basis for the stable photo‐responsive performance of the film system. In addition, the stretching vibration peak of N─H/O─H in the composite system exhibits an obvious regular shift. The characteristic peak of CDs/TEOA is at 3413 cm^−^^1^, while that of pure PVA is at 3428 cm^−^^1^. After compounding, this peak shifts to 3419 cm^−^^1^, which confirms the strong intermolecular hydrogen bonding between CDs/TEOA and PVA molecular chains and verifies the successful combination of CDs/TEOA with the PVA matrix [63].

**FIGURE 2:**
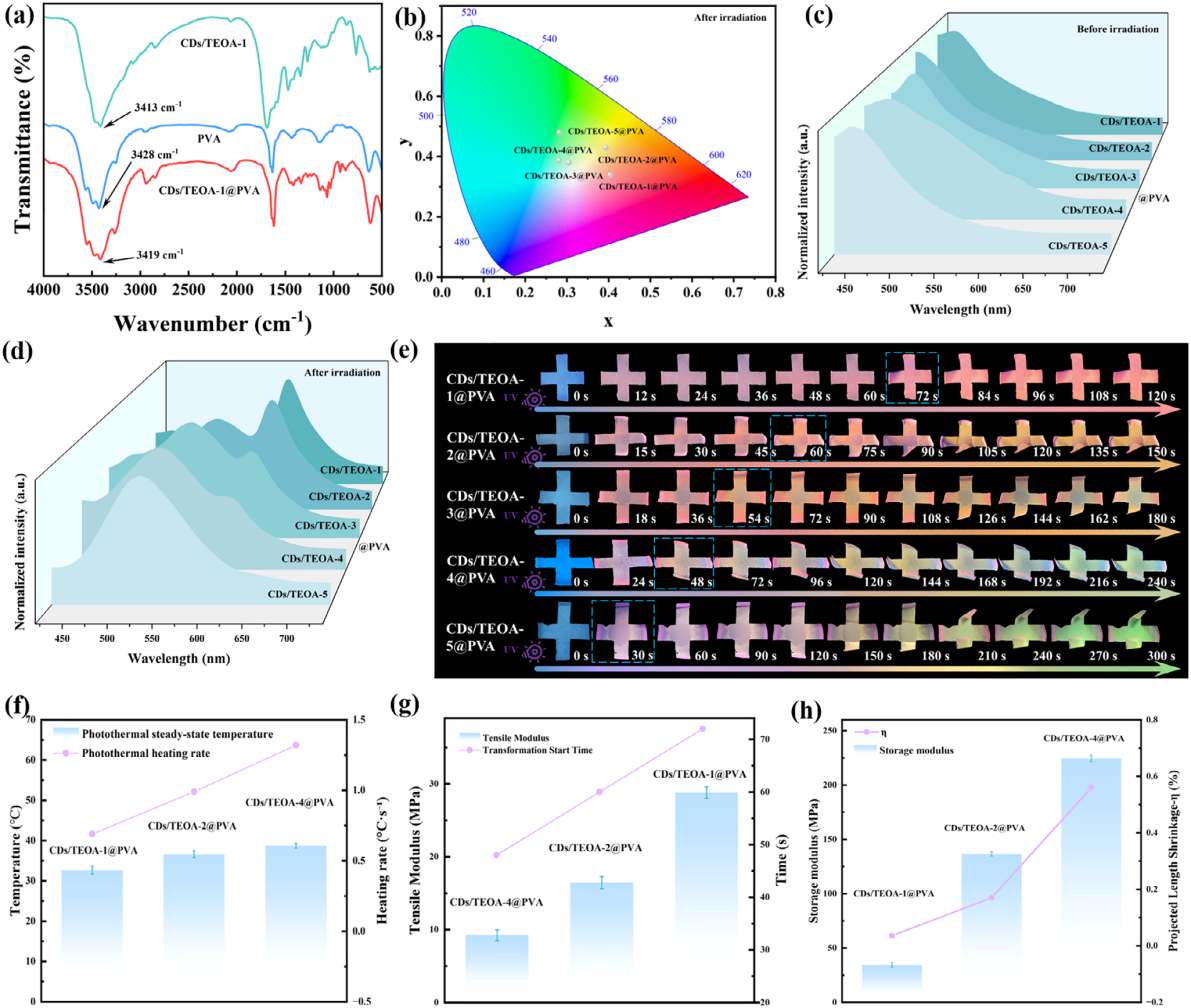
(a) FTIR spectra of PVA, CDs/TEOA‐1 and CDs/TEOA‐1@PVA; (b) CIE chromaticity coordinate diagrams of CDs/TEOA‐1–5@PVA after UV light irradiation; (c‐d) Normalized fluorescence emission spectra of CDs/TEOA‐1–5@PVA before and after UV light irradiation; (e) Optical photographs of the photoinduced chromism and bending deformation processes of CDs/TEOA‐1–5@PVA; (f) Photothermal steady‐state temperatures and heating rates of different CDs/TEOA@PVA composite films; (g) Tensile moduli and deformation onset times of different CDs/TEOA@PVA composite films; (h) Storage moduli and projected length shrinkage rates of different CDs/TEOA@PVA composite films.

We measured the UV–vis absorption spectra of CDs/TEOA@PVA composite films before and after 365 nm UV irradiation, and the results are presented in Figure S7. Upon continuous irradiation with 365 nm UV light, a series of new distinct characteristic absorption peaks appeared in these films, which were highly similar to the photo‐responsive absorption features of the pure CDs/TEOA system. The UV irradiation time required for the absorption peaks of the films to reach saturation gradually increased. From CDs/TEOA‐1@PVA to CDs/TEOA‐5@PVA, the saturation irradiation time was 120, 150, 180, 240, and 300 s, respectively, and the corresponding photo‐responsive fluorescence colors successively showed orange‐red, orange‐yellow, yellow, yellow‐green and green (Figure 2b). This variation trend is highly consistent with that of the pure CDs/TEOA system, confirming that the intrinsic photo‐responsive activity of CDs is fully retained after composite modification.

The time‐dependent fluorescence emission spectra of the composite films are shown in Figure 2c,d. Under 365 nm UV excitation, all CDs/TEOA@PVA composite films initially exhibited a strong characteristic blue fluorescence emission peak at 445 nm. With prolonged UV irradiation, the fluorescence intensity at 445 nm decreased steadily, while the intensities of newly generated emission peaks at 520 and 605 nm increased gradually. PVA‐based films with different dosages of the TEOA precursor ultimately achieved distinct steady‐state fluorescence emission and color evolution, realizing photo‐responsive multicolor fluorescence emission. Among them, CDs/TEOA‐1@PVA with the highest TEOA content showed the fastest photo‐response and reached a photo‐steady state after 120 s of continuous UV irradiation. Its emission peak at 445 nm attenuated significantly, whereas the peak at 605 nm continuously enhanced and became dominant (Figure S8a). Fluorescence lifetime results (Figure S9a–c, Table S5) revealed that within 0–120 s of irradiation, the fluorescence lifetime at 445 nm decreased from 4.92 to 1.70 ns, while those at 520 and 605 nm increased from 2.38 to 5.71 ns and from 2.57 to 3.32 ns, respectively. Combined with the CIE chromaticity diagram and digital fluorescence photographs (Figure S10a, Figure 3e), the fluorescence color gradually evolved from initial blue to orange‐red. For CDs/TEOA‐2@PVA, the dynamic fluorescence evolution was completed after 150 s of UV irradiation. The emission peak at 445 nm continuously declined, while the peaks at 520 and 605 nm increased synchronously with comparable final intensities and slight dominance of the 605 nm peak (Figure S8b). Accordingly, the fluorescence color changed gradually from blue to orange‐yellow (Figure S10b, Figure 3e), and the fluorescence lifetimes presented a consistent trend of short‐wavelength reduction and long‐wavelength enhancement (Figure S9d–f, Table S6).

**FIGURE 3:**
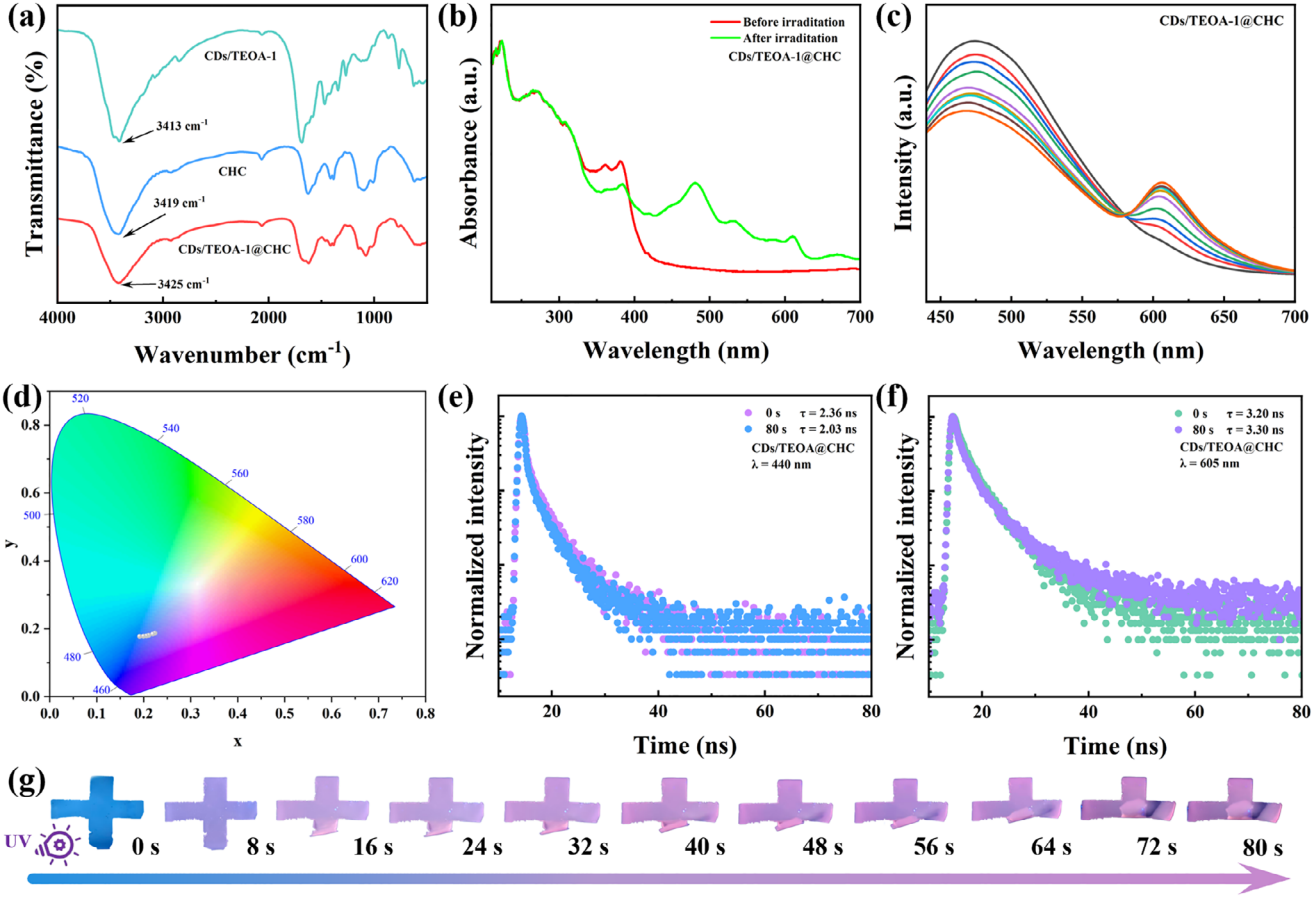
(a) FTIR spectra of CHC, CDs/TEOA‐1 and CDs/TEOA‐1@CHC composite films; (b) UV–vis absorption spectra of CDs/TEOA‐1@CHC composite film before and after UV light irradiation; (c) Fluorescence emission spectra of CDs/TEOA‐1@CHC composite film varying with UV light irradiation time; (d) CIE chromaticity coordinate diagram of CDs/TEOA‐1@CHC composite film; (e‐f) Fluorescence lifetime decay curves of CDs/TEOA‐1@CHC composite film at 440 nm (e) and 605 nm (f) after 0 and 80 s of irradiation, respectively; (g) Optical photographs of the photo‐induced bending deformation process of CDs/TEOA‐1@CHC composite film under continuous UV light irradiation for 0–80 s.

With the further reduction of TEOA dosage, CDs/TEOA‐3@PVA and CDs/TEOA‐4@PVA displayed similar fluorescence evolution behaviors. Both initially featured a dominant emission at 445 nm; upon irradiation, the short‐wavelength peak attenuated continuously while the two long‐wavelength peaks rose simultaneously, and the 520 nm emission finally became predominant (Figure S8c,d). These two films reached photo‐steady states after 180 and 240 s of irradiation, respectively, with different peak intensity differences, and their fluorescence colors converted to yellow and yellow‐green (Figure S10c,d, Figure 3e). Their fluorescence lifetimes dynamically responded synchronously to spectral evolution (Figure S9g–j, Tables S7 and S8). CDs/TEOA‐5@PVA with the lowest TEOA content exhibited unique fluorescence evolution. During 300 s of UV irradiation, the 445 nm emission continuously weakened, the 520 nm emission increased steadily, and the 605 nm emission first strengthened and then gradually faded. Eventually, the 520 nm emission peak completely dominated the spectrum (Figure S8e), and the fluorescence color changed progressively from blue to green (Figure S10e, Figure 4e). The variations in fluorescence lifetimes further confirmed the photoinduced dynamic fluorescence chromism of the films (Figure S9k–l, Table S9). Notably, the photoinduced fluorescence chromism of the composite films is intrinsically irreversible. Taking CDs/TEOA‐2@PVA as a representative sample, its orange fluorescence after UV‐induced chromism cannot recover to the initial blue state either after dark storage or upon secondary UV irradiation (Figure S11), confirming the irreversible conversion from naphthalimide to radical anions.To elucidate the core mechanism underlying the simultaneous photoinduced fluorescence discoloration and multicolor fluorescence performance of CDs/TEOA@PVA composite films, this paper compares the UV–vis absorption spectra of the composite films and pure CDs/TEOA aqueous solution before and after UV irradiation (Figures S3 and S7). The test results demonstrate that the spectral changes of the films after irradiation are highly consistent with those of the pure CDs/TEOA aqueous solution, and both show characteristic absorption peaks of naphthalimide radical anions in the visible light region [64, 65]. Meanwhile, no new characteristic absorption peaks appear in the FTIR spectra of CDs/TEOA@PVA composite films before and after UV irradiation (Figure S12), indicating that no new chemical bonds are generated during the irradiation process, thus excluding the possibility that photoinduced chromism is triggered by the cleavage and recombination of covalent bonds. Previous FTIR characterization has confirmed that the characteristic naphthalimide structure is successfully constructed in the composite system. Based on the above results, it is speculated that the photoinduced fluorescence discoloration of the films originates from naphthalimide radical anions produced by UV‐induced charge transfer; the multicolor fluorescence response is attributed to the difference in the content of naphthalimide structures on the surface of CDs in different samples, leading to a gradient change in the concentration of radical anions formed after irradiation. This inference can be preliminarily verified by the FTIR results: as shown in Figure S13, from CDs/TEOA‐1@PVA to CDs/TEOA‐5@PVA, the intensity of the characteristic stretching vibration peak at 1620 cm^−^^1^ ascribed to the O═C─N bond in the naphthalimide structure increases gradually [51].

**FIGURE 4:**
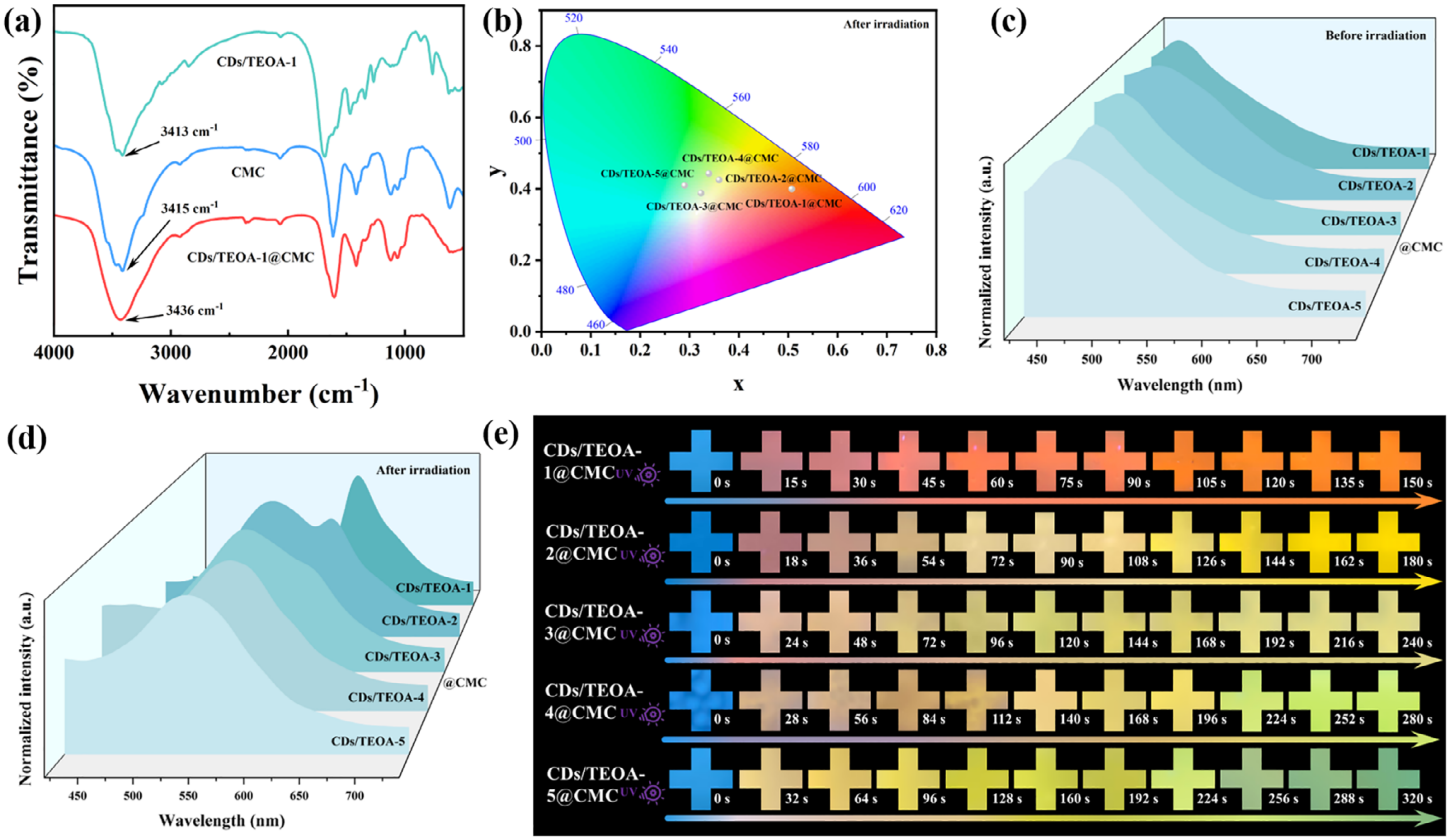
(a) FTIR spectra of CMC, CDs/TEOA‐1 and CDs/TEOA‐1@CMC; (b) CIE chromaticity coordinate diagrams of CDs/TEOA‐1–5@CMC after UV light irradiation; (c, d) Normalized fluorescence emission spectra of CDs/TEOA‐1–5@CMC before and after UV light irradiation; (e) Optical photographs of CDs/TEOA‐1–5@CMC composite films under continuous UV light irradiation.

Aiming at the remarkable macroscopic bending deformation of CDs/TEOA@PVA composite films under UV light irradiation, we systematically carried out a series of targeted characterization tests to clarify the underlying mechanism, and fully elucidated the correlation among the photothermal conversion properties, intrinsic dynamic mechanical properties and deformation behavior of the system. We monitored the temperature change of the films under 365 nm UV irradiation in real time via infrared thermal imaging. The results showed that all films in this series exhibited a significant photothermal temperature rise, demonstrating that this system can efficiently convert UV light energy into thermal energy, which provides the energy basis for the photoinduced deformation [66, 67]. On this basis, we further quantitatively characterized the photothermal performance of the films with different formulation ratios, and measured the steady‐state photothermal temperature and heating rate of the films from CDs/TEOA‐1@PVA to CDs/TEOA‐4@PVA. The results revealed that the steady‐state temperature of the films increased gradually from 32.7°C to 38.7°C, and the heating rate increased from 0.69°C/s to 1.32°C/s. The photothermal performance of the system changed regularly with the reactant ratio, and was highly correlated with the deformation behavior of the films (Figure 2f, Figure S14). From the perspective of excited state energy partitioning, absolute PLQY of CDs/TEOA‐1@PVA to ‐5@PVA was measured by integrating sphere at 400 nm excitation, yielding values of 5.53%, 4.09%, 2.81%, 2.38%, and 2.20%, respectively (Figure S15). The monotonic decrease with declining TEOA content demonstrates precise tunability of excited‐state energy distribution between radiative and non‐radiative channels. PLQY correlates negatively with photothermal temperature and heating rate, and with deformation onset time positively but deformation magnitude negatively, confirming enhanced non‐radiative relaxation as the core energy source for photothermal deformation. Moreover, PLQY only modulates energy allocation without altering the radical mechanism of fluorescence chromism, maintaining independent kinetic pathways and providing quantitative energy‐level support for temporal decoupling.

To accurately quantify the photoinduced deformation behavior of the films, we adopted the projection length shrinkage rate method based on top‐down orthographic imaging to perform continuous monitoring and quantitative characterization of the whole UV‐induced deformation process of the series of composite films, and obtained two core deformation parameters, namely the initial deformation trigger time and the maximum bending deformation degree. The test results demonstrate that the photoinduced deformation performance of the films from CDs/TEOA‐1@PVA to CDs/TEOA‐5@PVA shows a significant gradient optimization: the maximum projection length shrinkage rate increased monotonically from 3.6% to 64.1%, the initial deformation trigger time decreased markedly from 72 to 30 s, while the equilibrium time to reach the maximum deformation extended from 120 to 300 s (Figure S16, Figure 2e). Specifically, CDs/TEOA‐1@PVA exhibited macroscopically visible initial bending at 72 s and reached the maximum equilibrium shrinkage rate of 3.6% at 120 s. CDs/TEOA‐2@PVA, CDs/TEOA‐3@PVA, and CDs/TEOA‐4@PVA triggered initial deformation at 60, 54, and 48 s, and reached the maximum shrinkage rate of 18.6%, 23.9%, and 56.3% at 150, 180, and 240 s, respectively. CDs/TEOA‐5@PVA showed the fastest deformation response, with initial deformation occurring at only 30 s, and reached the maximum equilibrium shrinkage rate of 64.1% at 300 s (Figure S16a–e, Figure 2e). Further analysis revealed that the higher the steady‐state photothermal temperature and the faster the heating rate of the film, the shorter the initial deformation trigger time, which preliminarily established a direct correlation between the photothermal performance and the deformation response behavior of the system.

To further elucidate the regulation rule of the deformation behavior, we systematically characterized the key intrinsic dynamic mechanical property parameters of the series of CDs/TEOA@PVA composite films, including room‐temperature tensile modulus, loss factor tanδ, and storage modulus, and established a quantitative correlation between the mechanical properties and the deformation behavior. As shown in Figure 2g, the room‐temperature tensile modulus exhibits a strict positive correlation with the response time. The tensile modulus increased gradually from 9.379 MPa for CDs/TEOA‐4@PVA to 16.237 MPa, and finally reached 28.917 MPa for CDs/TEOA‐1@PVA, which verified the core law that “the lower the intrinsic modulus, the faster the deformation”. Materials with low modulus are more prone to elastic deformation under the same internal stress, which is macroscopically manifested as a faster response speed. In addition, the room‐temperature loss factor tanδ shows a negative correlation with the response time. The tanδ value increased from 0.295 to 0.51, and finally reached 0.55, reflecting the increase in the internal friction of the polymer chain segments. A higher tanδ indicates more sufficient energy dissipation of the chain segments during thermal motion and easier occurrence of segmental movement, which further accelerates the deformation response rate (Figure S17 and S18). Taken together, the above experiments and correlation analysis demonstrate that under the same matrix, the significant difference in the deformation response time of different CDs composite films is jointly determined by the intrinsic dynamic mechanical properties of the materials, with a clear quantitative correlation between the two.

In addition to the deformation onset time, this study also systematically analyzed the difference in the maximum bending deformation degree of the PVA‐based composite films. In terms of the maximum bending deformation degree, the room‐temperature storage modulus increased from 34.4 to 136.8 MPa, and finally reached 221.5 MPa for the films from CDs/TEOA‐1@PVA to CDs/TEOA‐4@PVA, which was positively proportional to the maximum bending deformation angle (Figure 2h and Figure S19). The essential reason lies in that a higher steady‐state photothermal temperature enables polymer chain segments to obtain more thermal energy and undergo larger‐amplitude motion; meanwhile, a higher intrinsic storage modulus endows the material with better elastic recovery ability and deformation bearing capacity, allowing it to withstand greater elastic deformation without fracture. Therefore, the maximum bending deformation degree is mainly determined by the amplitude of photothermally induced chain segment motion and the intrinsic elastic properties of the material.

Based on all the above experimental results from infrared thermal imaging, quantitative characterization of photothermal performance, quantitative analysis of deformation behavior and dynamic mechanical property tests, we have finally elucidated the photoinduced deformation mechanism of the CDs/TEOA@PVA composite films. With the same matrix, the differences in the deformation trigger time and the maximum bending deformation degree of the films are regulated by the synergistic effect of intrinsic dynamic mechanical properties, and the coupling effect of photothermally induced chain segment motion amplitude and intrinsic elastic properties, respectively. The essence of photoinduced deformation in this system is the enhancement of thermal motion of polymer chain segments driven by the photothermal effect. UV irradiation induces an overall temperature rise of the system, which provides the core energy for the thermal motion of PVA polymer chain segments. The elevated temperature continuously intensifies the thermal motion of the chain segments; when the motion capacity of the chain segments reaches the critical level for deformation, the internal stress stored during the material preparation process is fully released via chain segment motion, which forms the fundamental basis for the occurrence of photoinduced deformation behavior in this system. On this basis, the trigger time of the initial deformation of the films can be quantitatively regulated through the photothermal conversion efficiency and the intrinsic dynamic mechanical properties of the material. A higher steady‐state photothermal temperature and faster heating rate enable the material to reach the critical chain segment motion level required for deformation more rapidly; a lower tensile modulus and higher loss factor endow the chain segments with stronger intrinsic motion capacity. The synergistic effect of these two factors achieves precise control over the deformation response speed. Meanwhile, the ultimate achievable maximum bending deformation amplitude of the films is jointly determined by the amplitude of photothermally induced chain segment motion and the intrinsic elastic properties of the material. A higher photothermal temperature rise can drive the chain segments to achieve larger‐amplitude motion, while a higher storage modulus endows the material with superior elastic recovery and deformation bearing capacity. These two factors together determine the maximum deformation degree of the films.

### 2.5 Photo‐Responsive Performance of CDs/TEOA@Chc and CDs/TEOA@Cmc Composite Films

To further clarify the intrinsic mechanism of photoinduced chromism and photoinduced deformation and determine the coupling and decoupling rules between them, two polymer matrices (chitosan hydrochloride (CHC) and carboxymethyl chitosan (CMC)) were selected to construct corresponding control composite systems, and the structure and optical properties of the two composite systems were systematically characterized.

CDs/TEOA‐1 was blended with CHC solution to prepare CDs/TEOA‐1@CHC composite films. FTIR spectroscopy was used to compare and characterize the chemical structure and composite mechanism of pure CDs/TEOA‐1, pure CHC and the composite films. As shown in Figure 3a, the composite interaction rule of this system is highly consistent with that of the aforementioned PVA‐based composite films. FTIR results reveal that the naphthalimide structure in CDs/TEOA‐1 is completely retained. Meanwhile, the stretching vibration peak of N─H/O─H in the system shows a regular shift: this characteristic peak is located at 3413 cm^−^^1^ for CDs/TEOA‐1 and 3419 cm^−^^1^ for pure CHC, and shifts to 3428 cm^−^^1^ after compounding, indicating the effective compounding of the two components.

In the photo‐responsive performance test, two important experimental phenomena were observed: the film only exhibited basic photo‐fluorescent chromism without the multicolor fluorescence emission capability of the CDs/TEOA@PVA system, and meanwhile, the film still showed obvious macroscopic curling deformation under UV irradiation. As shown in Figure 3b, distinct new characteristic absorption peaks appeared after continuous irradiation for 80 s, confirming that UV irradiation successfully induced the generation of new luminescent species. The time‐dependent fluorescence emission spectra (Figure 3c) revealed that with the irradiation time prolonged from 0 to 80 s, the intensity of the emission peak at 440 nm continuously decreased, while a new fluorescence emission peak gradually emerged at 605 nm with steadily increased intensity, presenting the same photo‐fluorescent red‐shift characteristic as the PVA system, but the variation rate of the dual‐peak intensity ratio was relatively gentle. The CIE chromaticity diagram (Figure 3d) intuitively verified the evolution law of fluorescence color: with the increase of irradiation time, the fluorescence chromaticity coordinates of the film gradually shifted from the initial blue region to the long‐wavelength direction and finally stabilized in the magenta region. The fluorescence lifetime changes of the film before and after irradiation were characterized by time‐resolved fluorescence spectroscopy. The lifetime fitting results at the emission wavelength of 440 nm (Figure 3e and Table S10) showed that the average fluorescence lifetime of the film was 2.36 ns without irradiation and decreased to 2.03 ns after 80 s of UV irradiation, which was highly consistent with the attenuation trend of the fluorescence emission peak intensity at this position. The lifetime fitting results at the emission wavelength of 605 nm (Figure 3f) indicated that the initial average fluorescence lifetime of the film was 3.20 ns and slightly increased to 3.30 ns after 80 s of irradiation, effectively corroborating the dynamic evolution process of the film's fluorescence color.

Notably, during continuous UV irradiation for 0–80 s, the film exhibited significant macroscopic curling deformation consistent with that of the PVA system, and the free end of the film gradually curled upward with the prolongation of irradiation time (Figure 3g). The deformation law was quantitatively characterized by the projected length shrinkage method based on front‐top view imaging. The results showed that the shrinkage rate of the film increased monotonically with irradiation time: it was 0% at 0 s, rapidly reached 25.8% after 16 s of irradiation, and finally reached the maximum value of 71.3% at 80 s (Figure S20).

Next, using CMC as the polymer matrix, we prepared a full series of five CDs/TEOA@CMC composite films to investigate their photo‐responsive behavior. The structure and composite mechanism of the composite films were first characterized by FTIR spectroscopy. As shown in Figure 4a, the CDs/TEOA@CMC composite system exhibited similar structural variation trends to the PVA and CHC systems. Comparison of the FTIR spectra of pure CDs/TEOA, pure CMC, and the composite films revealed that no new characteristic absorption peaks can be observed in CDs/TEOA@CMC composite films, indicating that the intrinsic chemical structure of CDs/TEOA remains intact during the composite preparation process. Meanwhile, the characteristic stretching vibration peaks of O═C─N and C─N bonds corresponding to the naphthalimide ring structure were completely retained in the composite films, providing a core structural guarantee for the photo‐responsive performance of CDs/TEOA in the CMC film system. Notably, the stretching vibration peak of N─H/O─H was located at 3413 cm^−^^1^ for CDs/TEOA and 3415 cm^−^^1^ for CMC, and this peak shifted to 3436 cm^−^^1^ in the composite film, indicating the existence of intermolecular hydrogen bonding interactions between CDs/TEOA and CMC, and confirming the successful composite of CDs/TEOA with the CMC matrix.

On this basis, we systematically tested the photo‐responsive properties of this series of films, and the UV–vis absorption spectra are presented in Figure S21. Upon continuous 365 nm UV irradiation, new distinct characteristic absorption peaks emerged in the visible light range of 420–620 nm, which were highly consistent with the photo‐responsive absorption characteristics of CDs/TEOA. The saturated irradiation time of films from CDs/TEOA‐1@CMC to CDs/TEOA‐5@CMC was 150, 180, 240, 280, and 320 s, respectively, and the corresponding fluorescence colors sequentially presented orange‐red, orange‐yellow, yellow, yellow‐green, and green (Figure 4b).

Subsequently, the time‐dependent fluorescence emission spectra of the composite films were measured (Figure 4c,d). The results showed that under excitation with 365 nm UV light, all composite films exhibited a strong blue fluorescence emission peak at 445 nm in the initial state. With the prolongation of UV irradiation time, the intensity of the fluorescence emission peak at 445 nm continuously decreased, while the intensities of the emission peaks at 535 nm and 605 nm gradually increased. The films with different TEOA dosages finally showed differentiated steady‐state fluorescence emission characteristics and color changes, successfully realizing photo‐responsive multicolor fluorescence emission. Specifically, the CDs/TEOA‐1@CMC film had the fastest photo‐response rate and reached the photo‐stationary state after 150 s of UV irradiation, with the emission peak at 605 nm being dominant (Figure S22a), and the fluorescence color gradually changed from blue to orange‐red (Figure S23a, Figure 2e). Fluorescence lifetime tests confirmed the occurrence of this dynamic fluorescence discoloration process. During 0–150 s of irradiation, its fluorescence lifetime at 445 nm decreased from 2.10 to 1.62 ns, that at 535 nm increased from 2.64 to 4.98 ns, and that at 605 nm increased from 2.62 to 3.27 ns (Figure S24a–c, Table S11). The CDs/TEOA‐2@CMC film completed the dynamic fluorescence evolution after 180 s of UV irradiation, and finally the intensities of the emission peaks at 535 and 605 nm were similar with the former slightly higher (Figure S22b), and the fluorescence color changed from blue to orange‐yellow (Figure S23b, Figure 4e). During 0–180 s of irradiation, its fluorescence lifetime at 445 nm decreased from 2.59 to 1.90 ns, that at 535 nm increased from 4.13 to 8.03 ns, and that at 605 nm increased from 2.88 to 5.22 ns (Figure S24d–f, Table S12).

With further reduction of the TEOA dosage, the CDs/TEOA‐3@CMC and CDs/TEOA‐4@CMC films reached the photo‐stationary state after 240 and 280 s of UV irradiation, respectively. The emission peak at 445 nm continuously decreased, and the emission peak at 535 nm finally became dominant, with a smaller intensity difference between the two characteristic peaks for the former (Figure S22d,e). Their fluorescence colors changed from blue to yellow and yellow‐green, respectively (Figure S23c,d, Figure 4e). The fluorescence lifetimes of both films simultaneously showed a trend of attenuation at short wavelengths and increase at long wavelengths (Figure S24g–j, Tables S13 and S14). The CDs/TEOA‐5@CMC film with the lowest TEOA dosage required 320 s of UV irradiation to complete the fluorescence evolution. The emission peak at 605 nm first increased and then decreased until it disappeared, and the emission peak at 535 nm became dominant, with the fluorescence color changing from blue to green (Figure S22e and S23e, Figure 4e). The fluorescence lifetime also exhibited corresponding dynamic variation characteristics during irradiation (Figure S24k–l, Table S15).

Systematic comparison of the photo‐responsive behaviors of the three composite films reveals that the CDs/TEOA@PVA system can simultaneously realize the integrated multi‐dimensional photo‐functions of photo‐responsive fluorescence discoloration, photo‐responsive multicolor fluorescence emission and photoinduced deformation. In sharp contrast, the CDs/TEOA@CHC system only possesses the dual functions of photo‐responsive fluorescence discoloration and photoinduced deformation, while the CDs/TEOA@CMC system can only achieve photo‐responsive fluorescence discoloration and multicolor fluorescence emission. These results demonstrate that photoinduced chromism and photoinduced deformation can be fully decoupled in this system, with no inherent coupling between the two properties of photoinduced chromism and photoinduced deformation. Noteworthily, all films are prepared with a fixed volume of casting solution and identical effective film‐forming area. Cross‐sectional SEM characterization (Figure S25) confirms that all films feature dense and uniform cross‐sections free of obvious particle agglomeration, with thicknesses controlled within a narrow range of 25–28 µm and good consistency across samples of the same matrix. Therefore, the observed differences in photothermal temperature rise, deformation rate/magnitude, and fluorescence chromism kinetics all originate from the intrinsic photophysical and mechanical properties of the materials, rather than interference from inconsistent film thickness. This conclusion not only clarifies the mutual independence of the two photo‐responsive functional modules, but also provides core theoretical support for the subsequent in‐depth revelation of the mechanisms related to photo‐responsive fluorescence discoloration and photoinduced deformation in this work.

### 2.6 Photothermally Driven Photoinduced Deformation Mechanism

We found that CDs/TEOA@CHC and CDs/TEOA@PVA exhibited similar photoinduced deformation behavior, while the CDs/TEOA@CMC composite film did not show obvious photoinduced deformation. To clarify the cause of the deformation difference among the three types of films, we first monitored the real‐time temperature changes of the three composite films during UV irradiation via infrared thermal imaging. Under 365 nm UV irradiation, all three composite films showed a significant photothermal temperature rise (Figure S26), demonstrating that all of them can convert UV light energy into thermal energy, induce an overall temperature rise of the system, and provide a potential energy source for photoinduced deformation [68]. A non‐luminous blank sample without photoactive CDs was tested under identical irradiation conditions to exclude environmental heat accumulation from prolonged UV exposure. The blank sample showed only a negligible steady‐state temperature rise, far lower than that of all CDs/TEOA composite films, and no macroscopic deformation was observed throughout the test. This confirms that the observed photothermal heating and deformation originate from the intrinsic photothermal conversion of CDs/TEOA, with negligible contribution from the thermal radiation of the UV lamp.

On this basis, we systematically characterized the intrinsic dynamic mechanical properties of the three types of composite films with CMC, PVA and CHC matrices, and comparatively analyzed the differences in deformation behavior of different matrix systems in combination with the photothermal test results. At room temperature (25°C), the CDs/TEOA‐1@CMC composite film exhibits an ultrahigh storage modulus of 1150.4 MPa and a loss factor tanδ of only 0.162, indicating that its polymer chain segments have weak motion capacity and extremely strong intrinsic rigidity at room temperature (Figures S28 and S29). Combined with the photothermal test results, its steady‐state temperature under light irradiation is only 27.6°C, which only brings about a slight temperature rise and limited enhancement of chain segment motion. As a result, the internal stress cannot be released by overcoming the rigid resistance of the material, thus no macroscopic deformation is generated (Figure S26).

In sharp contrast, the CDs/TEOA‐1@PVA and CDs/TEOA‐1@CHC systems show significantly reduced room‐temperature storage modulus of 34.4 MPa and 21.4 MPa, respectively, along with a greatly elevated loss factor tanδ up to 0.295 and 1.525, demonstrating that their intrinsic chain segment motion capacity is far superior to that of the CMC matrix (Figures S28 and S29). Meanwhile, their steady‐state temperatures under irradiation reach 38.7°C and 36.0°C, respectively. The remarkable photothermal temperature rise drastically improves the motion capacity of the chain segments [69, 70], enabling the internal stress to be fully released by overcoming the resistance of the material, which in turn triggers significant macroscopic curling deformation (Figure S26). To further validate the photothermally driven deformation mechanism and rule out the non‐thermal deformation pathway induced by UV irradiation, we conducted additional isothermal control experiments on a precision hot stage. CDs/TEOA‐1@PVA and CDs/TEOA‐1@CHC films were heated to their respective steady‐state temperatures under UV irradiation (38.7°C and 36.0°C) and maintained isothermally for 300 s. No obvious curling or delamination deformation was observed in either film (Figure S27). Combined with the strong correlation between deformation behavior and the magnitude of photothermal temperature rise, this result excludes the potential deformation pathway directly triggered by pure photochemical processes, and confirms that the thermal effect is a necessary condition for deformation in the present system. Based on the above experimental data, it can be concluded that the on‐off switching difference of “no deformation for CMC while significant deformation for PVA/CHC” among the three types of matrix composite films originates from the different matching degrees between the intrinsic dynamic mechanical properties of the matrices and the enhancement level of chain segment motion induced by photothermal temperature rise.

### 2.7 Mechanism of Photoinduced Fluorescence Chromism

Through systematic characterization of the optical properties of the above three types of composite films, we confirmed that all three samples exhibit display photoinduced fluorescence chromism behavior. To verify the hypothesized mechanism that the photoinduced fluorescence chromism originates from the generation of naphthalimide radicals, we performed electron paramagnetic resonance (EPR) tests on the three types of composite films, with the results shown in Figure 5a–c. Before UV irradiation, no distinct characteristic EPR signal was detected for any of the three films. After UV irradiation, all samples exhibited a prominent characteristic signal at g = 2.0026, which is the signature peak of naphthalimide radical anions [71, 72]. This result directly confirms the generation of naphthalimide radical anions in the system upon UV irradiation, and provides decisive experimental evidence for the universal mechanism of photoinduced fluorescence chromism (Figure 5d). On the basis of clarifying the universal mechanism of photoinduced fluorescence chromism, we further elucidated the regulation mechanism of multicolor fluorescence emission. Analysis of the EPR spectra of the CDs/TEOA@CMC and CDs/TEOA@PVA systems (Figure 5b,c) revealed that, with the increase of TEOA dosage during the synthesis of CDs/TEOA, the intensity of the characteristic EPR signal at g = 2.0026 of the composite films after UV irradiation increased accordingly. This indicates that a larger amount of naphthalimide radical anions is generated in the system, which ultimately realizes the gradient regulation of the degree of photo‐responsive fluorescence chromism.

**FIGURE 5:**
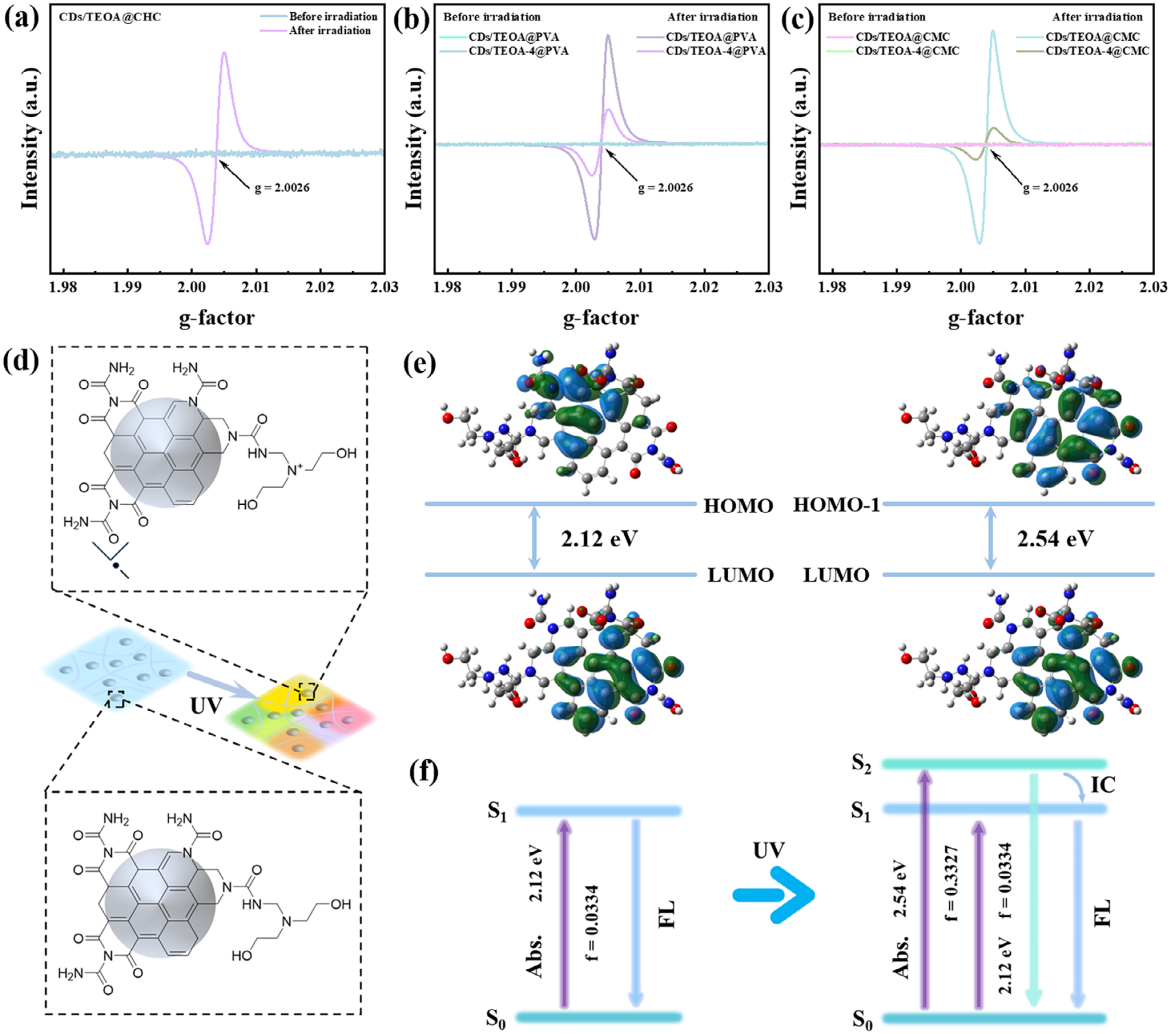
(a–c) EPR spectra of CDs/TEOA@CHC, CDs/TEOA@PVA and CDs/TEOA@CMC composite films before and after UV light irradiation; (d) Schematic diagram of the photoinduced fluorescence chromism mechanism of CDs/TEOA; (e) HOMO/LUMO electron cloud distributions and energy level differences of CDs/TEOA molecules before and after light irradiation (Model 1); (f) Schematic diagram of electron transition and fluorescence emission energy levels under UV light excitation.

To further elucidate the mechanisms governing the multicolor tunability, photo‐responsive dynamic fluorescence behavior and photoinduced deformation in this work, we selected two representative CDs/TEOA models (denoted as Model 1 and Model 2) to perform time‐dependent density functional theory (TD‐DFT) calculations at the B3LYP/6‐31G(d) theoretical level (Figure 5e). The calculated vertical excitation energies, corresponding wavelengths, oscillator strengths and main orbital contributions are summarized in Figure 5e, Figure S30 and Table S16. For both models, two low‐energy singlet excited states were identified as the main optical transition channels after UV irradiation (Figure 5f). The first excited state (S_1_) is located at approximately 585 nm with an extremely low oscillator strength (f≈0.03), while the second excited state (S_2_) is centered at around 488 nm with a prominent oscillator strength (f≈0.33). Molecular orbital analysis reveals that the high‐intensity S_2_ transition corresponds to the HOMO‐1‐LUMO excitation, whereas the low‐intensity S_1_ transition originates from the HOMO‐LUMO excitation. The large oscillator strength of the S_2_ state indicates that this transition is an optically allowed transition, with excellent spatial overlap between the HOMO‐1 and LUMO orbitals, which is consistent with the main absorption band (450 nm) observed in the experimental UV–vis spectrum (Figure S3). In contrast, the extremely low oscillator strength of the S_1_ state demonstrates that the HOMO‐LUMO transition is either a symmetry‐forbidden transition or has poor orbital overlap, which only presents as a weak shoulder peak in the absorption spectrum.

The weak S_1_ transition plays a critical role in the dynamic photoinduced fluorescence chromism behavior. The low oscillator strength indicates a long radiative lifetime of the S_1_ state [73]. This unique electronic structure allows UV light to be predominantly absorbed by the S_2_ state, followed by energy transfer to the S_1_ state via an ultrafast internal conversion process. The long‐lived S_1_ state can act as a “kinetic capacitor”, diverting the stored excitation energy into two independent pathways with inherently different time scales, thereby achieving orthogonal regulation of multiple photo‐responsive functions. Therefore, under UV light excitation, the CDs are preferentially excited to the S_2_ state (due to its large absorption cross section), and then relax to the S_1_ state through an ultrafast internal conversion process. The population of the long‐lived S_1_ state has sufficient time to undergo non‐radiative relaxation, which is conducive to the enhancement of the photothermal effect [73]. Such non‐radiative relaxation alters the relative intensity of the multiple emission peaks, leading to a gradual red shift of the fluorescence emission under continuous UV irradiation, which is exactly the observed dynamic behavior of photoinduced fluorescence chromism in our experiments.

The discrepancy between the two CDs/TEOA models further elucidates the multicolor emission characteristics. The S_1_ emission wavelengths of Model 1 and Model 2 (differing in the number of surface functional groups) are 584.5 and 590.4 nm, respectively, corresponding to a red shift of approximately 6 nm. This indicates that even minor changes in the CDs structure can effectively regulate the number of radicals, and thus tune the HOMO–LUMO energy gap, leading to the variation of the photo‐responsive fluorescence color. By systematically tuning the feed ratio during the synthesis process to regulate the number of radicals, we obtained a series of CDs structures with continuously gradient HOMO–LUMO energy gaps, thus realizing the broad‐range multicolor emission from blue to red light observed in our experiments [74]. Combined with the structural characterization results, the gradient change of radical concentration under UV irradiation essentially originates from the modification effect of TEOA on the surface structure of CDs. As a precursor, TEOA adjusts the relative content and electronic environment of naphthalimide functional groups on the CDs surface, which further determines the yield of radical anions generated under photoexcitation, and finally realizes the gradient regulation of fluorescence emission color [75].

Meanwhile, the non‐radiative decay of the S_1_ state generates the photothermal effect, and achieves significant heat accumulation by virtue of its long‐lived characteristic [76]. The accumulated heat raises the local temperature of the composite material, allowing the polymer chain segments to obtain thermal energy and release internal stress via segmental motion, which ultimately triggers the macroscopic bending of the material, namely the photoinduced deformation behavior. The temporal decoupling between photoinduced chromism and photoinduced deformation in this system is essentially attributed to the inherent rate difference between intramolecular conformational relaxation and polymer chain segmental relaxation. The time scales of these two processes differ by two orders of magnitude, enabling their spontaneous separation without any external intervention.

The TD‐DFT calculations further refine and complement the mechanism of dynamic photoinduced fluorescence chromism: the S_1_ transition endows the system with a long excited‐state lifetime, which provides the prerequisite for the non‐radiative relaxation required for the photo‐responsive dynamic fluorescence behavior; while the structure‐sensitive HOMO–LUMO energy gap governs the feed ratio‐dependent multicolor emission. These two intrinsic properties, originating from the electronic structure of the CDs, are the key to realizing the integration of the three core functions of multicolor luminescence, photoinduced chromism, and photoinduced deformation in this composite system.

### 2.8 Multi‐Dimensional Dynamic Information Encryption and Three‐Dimensional Anti‐Counterfeiting Applications

The photochromic and photoinduced deformation properties of the films prepared in this study enable dynamic multi‐modal information encryption. Using mask‐assisted UV writing technology, a heart‐shaped four‐leaf clover pattern was constructed from four different CDs/TEOA@CMC films (the four heart‐shaped regions correspond to CDs/TEOA‐1@CMC, CDs/TEOA‐2@CMC, CDs/TEOA‐3@CMC and CDs/TEOA‐4@CMC, respectively). Patterns with different fluorescent color combinations can be engraved within 320 s, and the heart‐shaped four‐leaf clover pattern exhibits a distinct fluorescence color transition behavior under controlled UV irradiation (Figure 6a). In addition to the time‐dependent fluorescence color‐changing patterns, we further integrated the CDs/TEOA@PVA series films with CDs/TEOA@CHC/CMC to form a 2 × 2 matrix, and designed a multi‐modal information encryption system (Figure 6b). This system adopts a double‐layer composite structure design, where the upper film is only fixed at the edges and can deform and peel off under UV irradiation, while the lower film is fully fixed on the glass substrate throughout the process. Specifically, for region ①, the upper layer is CDs/TEOA‐4@PVA and the corresponding lower layer is CDs/TEOA‐4@CMC; for region ②, the upper layer is CDs/TEOA‐3@PVA and the lower layer is CDs/TEOA‐3@CMC; for region ③, both the upper and lower layers are CDs/TEOA‐1@CHC; for region ④, the upper layer is CDs/TEOA‐1@PVA and the corresponding lower layer is CDs/TEOA‐1@CMC. During continuous UV irradiation, the upper layer regions are gradually peeled off, and the lower film patterned by mask‐assisted photolithography reveals the characters “NUFQ” with different fluorescent colors. These displayed characters are camouflaged encrypted information, which must be interpreted following a specific decoding method to obtain the correct information “QFNU”. Specifically, based on the difference in the photoinduced deformation response time of different films, the sequential order of film deformation and peeling is used as the basis for the alphabetical sorting of the correct information. The first peeled region corresponds to the first letter of the valid information, and the subsequent letters are determined in turn according to the time sequence of deformation. Furthermore, a letter can be output only when the final fluorescence color of the lower layer region matches that of the corresponding upper layer region. Based on the photophysical properties of the above multi‐responsive deformable films, a three‐dimensional stereoscopic anti‐counterfeiting system was constructed in this chapter. This system uses three orthogonal faces of a cube as the carriers for information storage and presentation, with each face integrated with a 3 × 3 information coding matrix (Figure 6c). The three orthogonal faces are each matched with one type of light‐responsive deformable film: the L‐face, T‐face and R‐face correspond to CDs/TEOA‐5@PVA, CDs/TEOA‐2@PVA and CDs/TEOA‐1@CHC films, respectively. Under UV irradiation, the three films show significant differences in macroscopic deformation rate: the film on the R‐face has the fastest deformation response, followed by that on the L‐face, while the film on the T‐face has the slowest deformation rate (Figure 6d). The fluorescence coding of this system exhibits a significant UV irradiation time dependence. At 0 s of UV irradiation, the L, T and R faces all show blue fluorescence. After 60 s of UV irradiation, the fluorescence colors of the L, T and R faces all change significantly; decryption against the A‐Z alphabet color block coding map (Figure 6e) yields the corresponding initial coding information at this time point: L_1_‐RFM, T_1_‐OVQ, R_1_‐CXZ. When the UV irradiation duration is extended to 320 s, the fluorescence colors of the L, T and R faces change again, and the corresponding coding information is synchronously updated to L_2_‐KBP, T_2_‐YNW, R_2_‐HAG. Among them, the correct information corresponding to the combined code LTR_1_ is ROC, obtained by splicing the first letter of each initial coding group; the correct information corresponding to the combined code LTR_2_ is BNA, obtained by splicing the second letter of each updated coding group. Only by sequentially splicing the coding information corresponding to the above LTR_1_ and LTR_2_ according to the completion sequence of macroscopic deformation of the deformable film on each face can the final complete key information CARBON of this three‐dimensional anti‐counterfeiting system be decrypted (Figure 6f). The dual response characteristics of the material, namely photoinduced multicolor fluorescence chromism and macroscopic deformation, break through the dimensional limitation of traditional encryption systems based on single fluorescence chromism, and provide a brand‐new design concept for multi‐dimensional and multi‐level dynamic information encryption.

**FIGURE 6:**
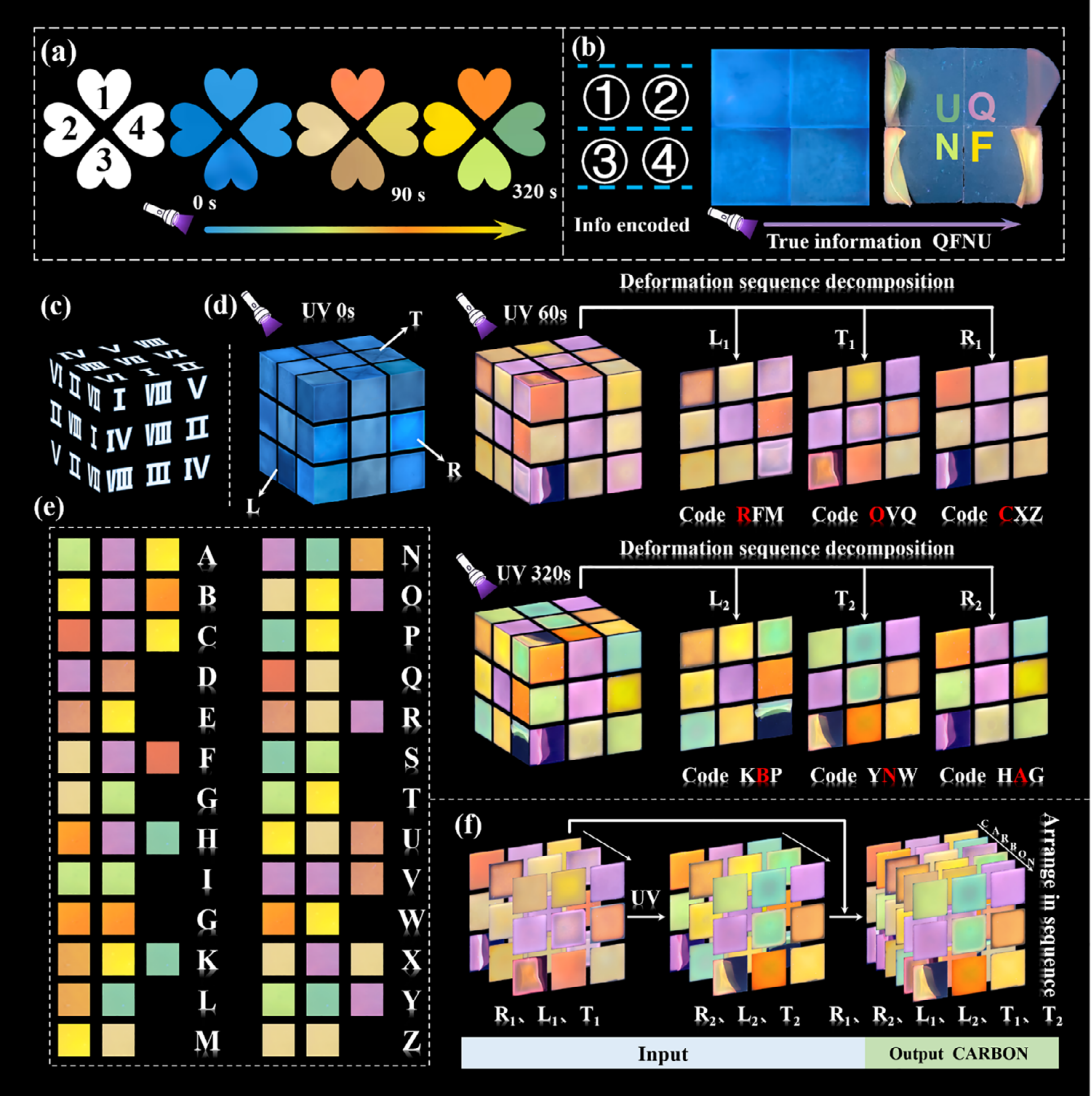
Multi‐dimensional information encryption applications based on CDs/TEOA composite films (a) Dynamic color change process of the four‐leaf clover pattern with UV irradiation time; (b) Demonstration of 2 × 2 array‐based information encryption and decryption; (c) Structural design of the 3D Rubik's cube‐like encryption carrier (I‐V correspond to CDs/TEOA‐1–5@CMC, VI corresponds to CDs/TEOA‐2@PVA, VII corresponds to CDs/TEOA‐5@PVA, and VIII corresponds to CDs/TEOA‐1@CHC); (d) Color change and deformation process, as well as deformation sequence decomposition of the 3D Rubik's cube at different UV irradiation durations; (e) Color coding‐based alphabet codebook; (f) Working workflow of the color‐deformation‐time multi‐dimensional.

## 3 Conclusions

To address the critical bottlenecks of traditional light‐driven 4D smart materials—high coupling between photoinduced deformation and photo‐responsive multicolor fluorescence that hinders independent regulation, as well as reusable and easily decrypted/copied dynamic encryption systems, this study carried out systematic innovative research based on the intrinsic excited‐state electronic structure of CDs. For the first time, we proposed and verified a novel energy regulation strategy using the S_1_ transition with weak oscillator strength and long excited‐state lifetime in naphthalimide‐functionalized CDs as a “kinetic capacitor”. The long lifetime of the S_1_ state enables dual‐channel programmable splitting of excited‐state energy: multicolor fluorescence emission via radiative relaxation, and macroscopic deformation driven by the photothermal effect via non‐radiative relaxation. This strategy achieves temporal kinetic decoupling of luminescence and deformation, breaking the constraint of fully synchronized dual responses in traditional coupled systems and providing a new paradigm for single‐source multi‐response regulation. By adjusting the TEOA feeding ratio to precisely tailor the HOMO‐LUMO energy gap of CDs, full‐spectrum gradient multicolor fluorescence emission from blue to orange‐red was achieved. Meanwhile, an independent regulation method for photothermally driven photoinduced deformation was established. Using PVA as the suitable matrix, the composite film realized the precise temporal decoupling of multicolor photoinduced chromism and photoinduced deformation for the first time, completing triple‐response 4D dynamic integration with irreversible characteristics. On this basis, a multi‐dimensional coupled key system integrating three‐dimensional spatial structure, fluorescence wavelength, light irradiation time, and deformation sequence was constructed relying on the material's time‐differentiated response and irreversible photo‐responsive properties. This system achieved high‐security one‐time information encryption, breaking through the dimensional and security bottlenecks of traditional encryption systems. It provides a new methodological support and technical pathway for the modular design of 4D smart materials and high‐end anti‐counterfeiting applications.

## Author Contributions

**Xiaoyu Xu**: data curation. **Meichen Meng**: formal analysis, writing – original draft. **Mingbo Yue**: data curation. **Chang Liu**: formal analysis, funding acquisition. **Qiang Fu**: data curation, formal analysis, writing – original draft, writing – review and editing, project administration, funding acquisition. **Zhimeng Ma**: data curation. **Jianye Zhang**: writing – original draft, data curation.

## Conflicts of Interest

The authors declare no conflicts of interest.

## Supporting information




**Supporting File**: advs76955‐sup‐0001‐SuppMat.docx.

## Data Availability

The data that support the findings of this study are available from the corresponding author upon reasonable request.
