# Supplementary material for: Temporal Decoupling: A New Paradigm of 4D Photo‐Responsive Carbon Dots Films With Modular Regulation of Color and Deformation
Source: Adv Sci (Weinh). 2026 Aug 6;13(60):e76955. doi: 10.1002/advs.76955 (PMC13445318; doi:10.1002/advs.76955)
Supplement: Supplementary file 1 [file ADVS-13-e76955-s001.docx]

**Supplementary Materials**

**Temporal Decoupling: A New Paradigm of 4D Photo-responsive Carbon Dots Films with Modular Regulation of Color and Deformation**

Jianye Zhang,^1[c]^ Meichen Meng,^1[a]^ Zhimeng Ma,^[c]^ Mingbo Yue,^[a]^ Xiaoyu Xu,^[b]^ Chang Liu,^*[b]^ Qiang Fu^*[ac]^

a.School of Chemistry and Chemical Engineering, Qufu Normal University, Qufu, Shandong, China

b.Shandong Agricultural University, College of Chemistry and Material Science, Taian, Shandong, China

c.College of Engineering, Qufu Normal University, Rizhao, Shandong, China

**S1. Supplementary Materials and Characterization Materials**

1,4,5,8-Naphthalenetetracarboxylic acid (purity 95%), urea (purity 99%) and chitosan hydrochloride (CHC) were purchased from Macklin Biochemical Technology Co., Ltd., Shanghai, China. Triethanolamine (TEOA) and N,N-dimethylformamide (DMF) were obtained from Tianjin Fuyu Fine Chemical Co., Ltd., Tianjin, China. Carboxymethyl chitosan (CMC) (BR, water-soluble) was bought from Shanghai Yien Chemical Technology Co., Ltd. Polyvinyl alcohol (PVA) was supplied by Sinopharm Chemical Reagent Co., Ltd., Shanghai, China. All reagents were of analytical grade and used as received without further purification. Deionized water was used throughout all experiments.

**Characterization**

The MinFlex600 X-ray diffractometer was used to measure the X-ray diffraction (XRD) patterns in the range of 10 to 60 degrees at room temperature. The scanning speed was 20°/min. The TEM-2100 was used to capture transmission electron microscope (TEM) images. Scanning electron microscopy (SEM) images were acquired using a Regulus 8100 scanning electron microscope. The Fourier transform infrared spectra (FTIR) were analyzed by using a Nicolet iS20spectrometer. A HORIBA LabRA laser confocal microscopic Raman spectrometer (excited by a 532 nm laser) was used for the Raman spectroscopy. The X-ray photoelectron spectroscopy (XPS) spectra were obtained using the X-ray photoelectron spectrometer (AXIS SUPRA+, SHIMADZU). The UV-vis absorption spectra were scanned by using a Lambda 1050 UV-Visible-near infrared spectrophotometer. The fluorescence spectra, fluorescence lifetime and photoluminescence quantum yields (PLQY) were measured on the steady-state and transient fluorescence spectrometer (Edinburgh FLS1000, UK) with a fixed excitation wavelength of 400 nm. Electron paramagnetic resonance spectroscopy was measured by paramagnetic resonance spectrometer (Bruker A300). Tensile modulus was tested using a CMT5504 microcomputer-controlled electronic universal testing machine. Frequency sweep tests were also carried out on the same CMT5504 instrument, where the storage modulus and loss factor (tanδ) of the samples were measured at room temperature under a frequency of 0.1 Hz. Real-time surface temperature distribution and temperature change curves of the samples during UV irradiation were recorded using a FLIR E8 Pro infrared thermal imager. The UV lamp used in the experiments has a power of 120 W with a wavelength of 365 nm, and the distance between the UV lamp and the sample surface is fixed at 8 cm.

**S2. Synthesis of CDs/TEOA and CDs/TEOA Aqueous Solutions with Different Ratios**

0.3 g of urea, 0.1 g of 1,4,5,8-naphthalenetetracarboxylic acid, and different volumes of TEOA (250, 200, 180, 150, 120 μL) were placed together in a 100 mL beaker. Subsequently, 8 mL of deionized water and 2 mL of N,N-dimethylformamide were added to the system, followed by ultrasonic treatment until all components were thoroughly and uniformly mixed, to prepare 5 groups of precursor mixtures with different TEOA ratios. The beaker was kept open and placed in a 700 W household microwave oven, which was operated in defrost mode (actual output power of the equipment under this mode: 294 W) for continuous microwave heating for 10 min. After the reaction was completed, the reaction system was left to stand and naturally cool to room temperature, and the corresponding crude products were collected, which were named CDs/TEOA-1, CDs/TEOA-2, CDs/TEOA-3, CDs/TEOA-4, and CDs/TEOA-5 in descending order of the TEOA feeding volume. Finally, The obtained crude product was dispersed in deionized water at a solid-liquid ratio of 1:50 (g:mL) to prepare a suspension, which was purified by centrifugation at 8000 rpm for 10 min per cycle. The supernatant was discarded after each centrifugation. This centrifugal washing procedure was repeated three times until no obvious fluorescence signal could be detected in the supernatant. The CDs precipitate at the bottom of the centrifuge tube was collected and vacuum-dried at 60 °C to yield CDs/TEOA-1~CDs/TEOA-5. 20 mg of each purified CDs/TEOA-1, CDs/TEOA-2, CDs/TEOA-3, CDs/TEOA-4, and CDs/TEOA-5 was added to 2 mL of deionized water separately, followed by ultrasonic treatment at room temperature for 2 h to achieve sufficient and uniform dispersion of CDs/TEOA, yielding a series of CDs/TEOA aqueous solutions with different ratios.

**S3. Preparation of CMC Solution**

0.3 g of CMC was weighed and dissolved in 10 mL of deionized water. After mixing, the mixture was left to stand for 1 h to obtain the CMC solution.

**S4. Preparation of CHC Solution**

0.3 g of CHC was dissolved in 10 mL of deionized water. The mixture was homogenized and left to stand for 1 h to obtain the CHC solution.

**S5. Preparation of PVA Solution**

0.6 g of PVA was weighed and thoroughly pre-mixed with 20 mL of deionized water, then the entire mixture was transferred to a 50 mL round-bottom flask. The flask was fixed in a constant-temperature oil bath device with dimethyl silicone oil as the heat transfer medium. The system was set at a temperature of 90 °C and a mechanical stirring speed of 700 r/min, and heated and stirred continuously under these conditions for 10 h. A homogeneous, transparent PVA matrix solution with good stability was obtained once the PVA was completely dissolved and no agglomerates visible to the naked eye remained in the system.

**S6. Preparation of CDs/TEOA-1@CHC Composite Film**

For the composite film, 18.5 mg of CDs/TEOA-1 was mixed with 1.5 mL of CHC solution, followed by ultrasonic treatment for 2 h to achieve uniform mixing. The above mixed solution was uniformly dropped onto the surface of a clean cover glass, and naturally dried at room temperature for 10 h at a constant ambient temperature of 25 ± 2 °C. The CDs/TEOA-1@CHC composite film was obtained after the solvent was completely volatilized and the film layer was thoroughly dried.

**S7. Preparation of CDs/TEOA@CMC Composite Films**

18.5 mg of each of the as-prepared CDs/TEOA-1, CDs/TEOA-2, CDs/TEOA-3, CDs/TEOA-4, and CDs/TEOA-5 samples was mixed with 1.5 mL of CMC solution one by one, and ultrasonicated at room temperature for 2 h to uniformly disperse CDs/TEOA in the CMC solution, obtaining 5 groups of homogeneous CDs/TEOA-polymer casting solutions. Each group of casting solution was uniformly drop-coated onto the surface of a clean glass slide substrate respectively, and left to dry naturally at room temperature for 10 h at a constant ambient temperature of 25 ± 2 °C. After the solvent was completely volatilized and the film layer was fully cured and formed, the corresponding CDs-doped composite films were obtained, which were named CDs/TEOA-1@CMC, CDs/TEOA-2@CMC, CDs/TEOA-3@CMC, CDs/TEOA-4@CMC, and CDs/TEOA-5@CMC in sequence.

**S8. Preparation of CDs/TEOA@PVA Composite Films**

18.5 mg of each of the CDs/TEOA-1, CDs/TEOA-2, CDs/TEOA-3, CDs/TEOA-4, and CDs/TEOA-5 samples was weighed and fully mixed with 1.5 mL of PVA solution respectively, and ultrasonicated at room temperature for 2 h to obtain 5 groups of homogeneous casting systems. Each group of casting solution was uniformly coated onto the surface of a pre-treated clean cover glass substrate respectively, and left to air-dry naturally at room temperature for 10 h at a constant ambient temperature of 25 ± 2 °C until the solvent was completely volatilized and the film structure was stably formed. Finally, a series of CDs/TEOA@PVA composite films were obtained, which were named CDs/TEOA-1@PVA, CDs/TEOA-2@PVA, CDs/TEOA-3@PVA, CDs/TEOA-4@PVA, and CDs/TEOA-5@PVA in sequence.

**S9. Average fluorescence lifetime**

The average fluorescence lifetimes were calculated according to the following Equation.

$$\tau_{avg}=\frac{\sum A_{i}\tau_{i}^{2}}{\sum A_{i}\tau_{i}}$$

are components of fluorescence lifetime and corresponding fitting parameters, respectively.

**S10. Projected Length Shrinkage Ratio**

The deformation degree of the photoactuated film was quantified by the projected length shrinkage ratio, which is defined as the ratio of the reduction in the horizontal projected length of the film after light-induced deformation to its initial flat length. The length was calculated through image analysis using ImageJ software.

The calculation formula for the projected length shrinkage ratio η is as follows:

$$\eta=\frac{L_{0}-L}{L_{0}}$$

η is the horizontal projected length shrinkage ratio, expressed in %; L0 is the initial actual length of the film in the flat state, in mm; L is the horizontal projected length of the film after light-induced bending, in mm.

**S11. Theoretical Calculations**

The main electronic transitions of CDs/TEOA with different reactant ratios before and after UV irradiation were characterized based on computational calculations. Calculation details: Time-dependent density functional theory (TD-DFT) calculations were performed at the B3LYP/6-31G(d) theoretical level. All calculations were completed using the Gaussian 09 software package. The excited-state properties were calculated via the TD-DFT method, and the geometric structure of the excited states was optimized using the same method as that for the ground state.

**S12. Supplementary Figures and Tables**

**Figure S1.** TEM images of CDs/TEOA with different ratios. Inset: TEM i mage and corresponding particle size distribution. (a) CDs/TEOA-1; (b) CDs/TEOA-2; (c) CDs/TEOA-3; (d) CDs/TEOA-4; (e) CDs/TEOA-5

**Figure S2.** Raman spectra of CDs/TEOA-1 to CDs/TEOA-5

**Figure S3.** UV-vis absorption spectra of CDs/TEOA with different ratios before and after UV irradiation. (a) CDs/TEOA-1; (b) CDs/TEOA-2; (c) CDs/TEOA-3; (d) CDs/TEOA-4; (e) CDs/TEOA-5

**Figure S4.** Optical photographs and fluorescence photographs of CDs/TEOA with different ratios before and after continuous UV irradiation. (a) Optical photographs before irradiation; (b) Fluorescence photographs before irradiation; (c) Optical photographs after irradiation; (d) Fluorescence photographs after irradiation

**Figure S5.** Schematic diagram of the time-dependent fluorescence color change of CDs/TEOA with different ratios under continuous UV irradiation. (a) CDs/TEOA-1; (b) CDs/TEOA-2; (c) CDs/TEOA-3; (d) CDs/TEOA-4; (e) CDs/TEOA-5

**Figure S6.** CIE chromaticity diagrams of CDs/TEOA with different ratios under continuous UV irradiation (Inset: fluorescence switching cycle process of aqueous solutions of CDs/TEOA with different ratios). (a) CDs/TEOA-1; (b) CDs/TEOA-2; (c) CDs/TEOA-3; (d) CDs/TEOA-4; (e) CDs/TEOA-5

**Figure S7.** UV-vis absorption spectra of CDs/TEOA@PVA series composite films before and after UV irradiation. (a) CDs/TEOA-1@PVA; (b) CDs/TEOA-2@PVA; (c) CDs/TEOA-3@PVA; (d) CDs/TEOA-4@PVA; (e) CDs/TEOA-5@PVA

**Figure S8.** Fluorescence spectra of CDs/TEOA@PVA series composite films as a function of irradiation time. (a) CDs/TEOA-1@PVA; (b) CDs/TEOA-2@PVA; (c) CDs/TEOA-3@PVA; (d) CDs/TEOA-4@PVA; (e) CDs/TEOA-5@PVA

**Figure S9.** Fluorescence decay curves of the five CDs/TEOA@PVA composite films. (a–c) Fluorescence decay curves of CDs/TEOA-1@PVA under different irradiation times (λ = 445 (a), 520 (b) and 605 (c) nm); (d–f) Fluorescence decay curves of CDs/TEOA-2@PVA under different irradiation times (λ = 445 (d), 520 (e) and 605 (f) nm); (g–h) Fluorescence decay curves of CDs/TEOA-3@PVA under different irradiation times (λ = 445 (g) and 520 (h) nm); (i–j) Fluorescence decay curves of CDs/TEOA-4@PVA under different irradiation times (λ = 445 (i) and 520 (j) nm); (k–l) Fluorescence decay curves of CDs/TEOA-5@PVA under different irradiation times (λ = 445 (k) and 520 (l) nm)

**Figure S10.** CIE chromaticity diagrams of CDs/TEOA@PVA series composite films. (a) CDs/TEOA-1@PVA; (b) CDs/TEOA-2@PVA; (c) CDs/TEOA-3@PVA; (d) CDs/TEOA-4@PVA; (e) CDs/TEOA-5@PVA

**Figure S11.** Irreversible photoinduced chromism of CDs/TEOA-2@PVA film after dark storage and secondary UV irradiation

**Figure S12.** FTIR spectra of CDs/TEOA-1@PVA composite film before and after UV irradiation

**Figure S13.** FTIR spectra of different CDs/TEOA@PVA composite films

**Figure S14.** Photothermal heating curves of different CDs/TEOA@PVA composite films as a function of UV irradiation time

**Figure S15.** Absolute photoluminescence quantum yield (PLQY) spectra of CDs/TEOA-1@PVA to CDs/TEOA-5@PVA films under 400 nm excitation: (a) CDs/TEOA-1@PVA; (b) CDs/TEOA-2@PVA; (c) CDs/TEOA-3@PVA; (d) CDs/TEOA-4@PVA; (e) CDs/TEOA-5@PVA

**Figure S16.** Deformation degree of CDs/TEOA@PVA series films under continuous UV irradiation. (a) CDs/TEOA-1@PVA; (b) CDs/TEOA-2@PVA; (c) CDs/TEOA-3@PVA; (d) CDs/TEOA-4@PVA; (e) CDs/TEOA-5@PVA

**Figure S17.** Curves of loss factor (tanδ) versus frequency for different CDs/TEOA@PVA composite films

**Figure S18.** Comparison of loss factor and initial deformation time for CDs/TEOA@PVA series composite films

**Figure S19.** Curves of storage modulus versus frequency for different CDs/TEOA@PVA composite films

**Figure S20.** Deformation degree of CDs/TEOA-1@CHC under continuous UV irradiation

**Figure S21.** UV-vis absorption spectra of CDs/TEOA@PVA series composite films before and after UV irradiation. (a) CDs/TEOA-1@CMC; (b) CDs/TEOA-2@CMC; (c) CDs/TEOA-3@CMC; (d) CDs/TEOA-4@CMC; (e) CDs/TEOA-5@CMC

**Figure S22.** Fluorescence spectra of CDs/TEOA@CMC series composite films as a function of irradiation time. (a) CDs/TEOA-1@CMC; (b) CDs/TEOA-2@CMC; (c) CDs/TEOA-3@CMC; (d) CDs/TEOA-4@CMC; (e) CDs/TEOA-5@CMC

**Figure S23.** CIE chromaticity diagrams of CDs/TEOA@CHC series composite films. (a) CDs/TEOA-1@CMC; (b) CDs/TEOA-2@CMC; (c) CDs/TEOA-3@CMC; (d) CDs/TEOA-4@CMC; (e) CDs/TEOA-5@CMC

**Figure S24.** Fluorescence decay curves of the five CDs/TEOA@CMC composite films. (a–c) Fluorescence decay curves of CDs/TEOA-1@CMC under different irradiation times (λ = 445 (a), 535 (b) and 605 (c) nm); (d–f) Fluorescence decay curves of CDs/TEOA-2@CMC under different irradiation times (λ = 445 (d), 535 (e) and 605 (f) nm); (g–h) Fluorescence decay curves of CDs/TEOA-3@CMC under different irradiation times (λ = 445 (g) and 535 (h) nm); (i–j) Fluorescence decay curves of CDs/TEOA-4@CMC under different irradiation times (λ = 445 (i) and 535 (j) nm); (k–l) Fluorescence decay curves of CDs/TEOA-5@CMC under different irradiation times (λ = 445 (k) and 535 (l) nm)

**Figure S25.** Cross-sectional SEM images of different composite films: (a) CDs/TEOA-1@PVA, (b) CDs/TEOA-5@PVA, (c) CDs/TEOA-1@CHC, (d) CDs/TEOA-1@CMC, (e) CDs/TEOA-5@CMC (Scale bar: 20 μm)

**Figure S26.** Photothermal heating curves of composite films with different matrices and the blank control as a function of UV irradiation time

**Figure S27.** Morphologies of the composite films before and after isothermal treatment on a hot stage. (a). CDs/TEOA-1@PVA (left: initial state without heating; right: after isothermal holding at 38.7 °C for 300 s); (b). CDs/TEOA-1@CHC (left: initial state without heating; right: after isothermal holding at 36.0 °C for 300 s)

**Figure S28.** Curves of loss factor (tanδ) versus frequency for composite films with different matrices

**Figure S29.** Curves of storage modulus versus frequency for composite films with different matrices

**Figure S30.** Electron cloud distribution of frontier molecular orbitals and energy level gap of CDs/TEOA molecules (Model 2)

**Table S1.** Table of Relative Contents of C, N and O Elements for CDs/TEOA-1~5

| Samples | C (%) | N (%) | O (%) |
| --- | --- | --- | --- |
| CDs/TEOA-1 | 61.17 | 18.80 | 20.03 |
| CDs/TEOA-2 | 61.79 | 18.80 | 20.03 |
| CDs/TEOA-3 | 63.60 | 18.49 | 19.28 |
| CDs/TEOA-4 | 63.94 | 17.13 | 19.18 |
| CDs/TEOA-5 | 65.91 | 16.88 | 18.89 |

**Table S2.** The peak area ratio of chemical bonds in C 1s of CDs/TEOA

| Samples | C=C/C-C (%) | C-O (%) | C-N (%) | C=O (%) |
| --- | --- | --- | --- | --- |
| CDs/TEOA-1 | 63.74 | 7.33 | 6.34 | 22.59 |
| CDs/TEOA-2 | 65.01 | 7.16 | 5.42 | 22.41 |
| CDs/TEOA-3 | 66.44 | 7.05 | 4.87 | 21.64 |
| CDs/TEOA-4 | 67.77 | 7.04 | 4.84 | 20.35 |
| CDs/TEOA-5 | 68.52 | 6.94 | 4.73 | 19.82 |

**Table S3.** The peak area ratio of chemical bonds in N 1s of CDs/TEOA

| Samples | N-H (%) | C-N (%) |
| --- | --- | --- |
| CDs/TEOA-1 | 89.79 | 10.21 |
| CDs/TEOA-2 | 85.24 | 14.76 |
| CDs/TEOA-3 | 82.76 | 17.24 |
| CDs/TEOA-4 | 81.97 | 18.03 |
| CDs/TEOA-5 | 81.47 | 18.53 |

**Table S4.** The peak area ratio of chemical bonds in O 1s of CDs/TEOA

| Samples | C-O (%) | C=O (%) |
| --- | --- | --- |
| CDs/TEOA-1 | 21.47 | 78.53 |
| CDs/TEOA-2 | 20.81 | 79.19 |
| CDs/TEOA-3 | 19.09 | 80.91 |
| CDs/TEOA-4 | 18.96 | 81.04 |
| CDs/TEOA-5 | 17.38 | 82.62 |

**Table S5.** Average fluorescence lifetime of CDs/TEOA-1@PVA under different UV irradiation times

| λ (nm) | UV light exposure time (s) | A_1_ (%) | τ_1_ (ns) | A_2_ (%) | τ_2_ (ns) | <τ> (ns) |
| --- | --- | --- | --- | --- | --- | --- |
| 445 | 0 s | 1.1231 | 1.32267 | 0.04624 | 13.49082 | 4.92 |
|  | 80 s | 0.02588 | 8.55411 | 1.33566 | 0.5502 | 2.40 |
|  | 150 s | 1.22325 | 0.46953 | 0.03222 | 5.62197 | 1.70 |
| 520 | 0 s | 0.92351 | 0.71077 | 0.19915 | 3.82335 | 2.38 |
|  | 80 s | 0.89017 | 0.69531 | 0.3142 | 5.90517 | 4.60 |
|  | 150 s | 0.55636 | 0.83302 | 0.49782 | 6.41926 | 5.71 |
| 605 | 0 s | 1.06928 | 0.62394 | 0.25452 | 3.88972 | 2.57 |
|  | 80 s | 0.51684 | 1.75079 | 0.54325 | 3.7536 | 3.14 |
|  | 150 s | 0.47794 | 1.66087 | 0.56159 | 3.91412 | 3.32 |

**Table S6.** Average fluorescence lifetime of CDs/TEOA-2@PVA under different UV irradiation times

| λ (nm) | UV light exposure time (s) | A_1_ (%) | τ_1_ (ns) | A_2_ (%) | τ_2_ (ns) | <τ> (ns) |
| --- | --- | --- | --- | --- | --- | --- |
| 445 | 0 s | 1.17453 | 0.63206 | 0.16944 | 7.25882 | 4.76 |
|  | 90 s | 1.45313 | 0.59851 | 0.04771 | 5.95139 | 1.92 |
|  | 180 s | 0.59238 | 0.82678 | 0.42123 | 0.82678 | 0.83 |
| 520 | 0 s | 0.93408 | 0.8628 | 0.13889 | 4.43075 | 2.40 |
|  | 90 s | 1.49157 | 0.74499 | 0.23521 | 6.59482 | 4.15 |
|  | 180 s | 0.43018 | 1.54587 | 0.5997 | 8.12342 | 7.33 |
| 605 | 0 s | 0.89883 | 0.9903 | 0.18987 | 4.64208 | 2.81 |
|  | 90 s | 0.38734 | 2.18142 | 0.62115 | 4.37638 | 3.86 |
|  | 180 s | 0.68707 | 3.02138 | 0.36008 | 6.28946 | 4.73 |

**Table S7.** Average fluorescence lifetime of CDs/TEOA-3@PVA under different UV irradiation times

| λ (nm) | UV light exposure time (s) | A1 (%) | τ1 (ns) | A2 (%) | τ2 (ns) | <τ> (ns) |
| --- | --- | --- | --- | --- | --- | --- |
| 445 | 0 s | 0.97962 | 0.87822 | 0.28304 | 7.91054 | 5.96 |
|  | 120 s | 1.52809 | 0.73871 | 0.08999 | 7.47342 | 3.25 |
|  | 240 s | 1.08262 | 0.84543 | 0.09967 | 5.55076 | 2.62 |
| 520 | 0 s | 1.04698 | 0.94091 | 0.21209 | 5.22181 | 3.21 |
|  | 120 s | 0.6513 | 0.80612 | 0.3814 | 8.32725 | 7.26 |
|  | 240 s | 0.37305 | 1.3126 | 0.6086 | 8.92518 | 8.30 |

**Table S8.** Average fluorescence lifetime of CDs/TEOA-4@PVA under different UV irradiation times

| λ (nm) | UV light exposure time (s) | A1 (%) | τ1 (ns) | A2 (%) | τ2 (ns) | <τ> (ns) |
| --- | --- | --- | --- | --- | --- | --- |
| 445 | 0 s | 0.94425 | 0.90226 | 0.35092 | 8.18255 | 6.52 |
|  | 140 s | 1.42874 | 0.64033 | 0.08731 | 8.28283 | 4.01 |
|  | 280 s | 0.59202 | 0.67518 | 0.55632 | 0.67519 | 0.68 |
| 520 | 0 s | 0.80039 | 0.55778 | 0.21082 | 4.79841 | 3.50 |
|  | 140 s | 0.58441 | 0.90204 | 0.55711 | 8.6204 | 7.86 |
|  | 280 s | 0.2625 | 1.75747 | 0.71891 | 9.44178 | 8.95 |

**Table S9.** Average fluorescence lifetime of CDs/TEOA-5@PVA under different UV irradiation times

| λ (nm) | UV light exposure time (s) | A1 (%) | τ1 (ns) | A2 (%) | τ2 (ns) | <τ> (ns) |
| --- | --- | --- | --- | --- | --- | --- |
| 445 | 0 s | 1.25899 | 0.57063 | 0.24359 | 8.45522 | 6.42 |
|  | 160 s | 1.97905 | 0.50659 | 0.11082 | 8.09883 | 4.09 |
|  | 320 s | 1.03057 | 0.49805 | 0.04634 | 6.25758 | 2.58 |
| 520 | 0 s | 0.96097 | 0.7845 | 0.20294 | 5.74976 | 3.80 |
|  | 160 s | 0.71014 | 1.19926 | 0.42482 | 8.75846 | 7.35 |
|  | 320 s | 0.24449 | 2.42034 | 0.72471 | 10.58545 | 10.00 |

**Table S10.** Average fluorescence lifetime of CDs/TEOA-1@CHC under different UV irradiation times

| λ (nm) | UV light exposure time (s) | A_1_ (%) | τ_1_ (ns) | A_2_ (%) | τ_2_ (ns) | <τ> (ns) |
| --- | --- | --- | --- | --- | --- | --- |
| 445 | 0 | 1.04304 | 0.61114 | 0.17608 | 3.96465 | 2.36 |
|  | 80 | 1.20902 | 0.55137 | 0.1361 | 3.89687 | 2.03 |
| 605 | 0 | 0.72574 | 0.78984 | 0.42997 | 4.00467 | 3.20 |
|  | 80 | 0.54756 | 1.09751 | 0.46335 | 4.01213 | 3.30 |

**Table S11.** Average fluorescence lifetime of CDs/TEOA-1@CMC under different UV irradiation times

| λ (nm) | UV light exposure time (s) | A1 (%) | τ1 (ns) | A2 (%) | τ2 (ns) | <τ> (ns) |
| --- | --- | --- | --- | --- | --- | --- |
| 445 | 0 s | 0.92331 | 0.61082 | 0.07442 | 4.56704 | 2.10 |
|  | 60 s | 1.53029 | 0.57153 | 0.04997 | 5.68932 | 1.83 |
|  | 120 s | 1.42533 | 0.55967 | 0.04435 | 5.24325 | 1.62 |
| 535 | 0 s | 0.86827 | 0.971 | 0.17071 | 4.47317 | 2.64 |
|  | 60 s | 0.91724 | 0.88814 | 0.18063 | 5.99393 | 3.80 |
|  | 120 s | 0.88548 | 0.85925 | 0.34777 | 6.39407 | 4.98 |
| 605 | 0 s | 0.73655 | 0.78567 | 0.31984 | 3.55615 | 2.62 |
|  | 60 s | 0.4406 | 1.59192 | 0.59949 | 3.61301 | 3.12 |
|  | 120 s | 0.64923 | 2.16103 | 0.35414 | 4.28559 | 3.27 |

**Table S12.** Average fluorescence lifetime of CDs/TEOA-2@CMC under different UV irradiation times

| λ (nm) | UV light exposure time (s) | A1 (%) | τ1 (ns) | A2 (%) | τ2 (ns) | <τ> (ns) |
| --- | --- | --- | --- | --- | --- | --- |
| 445 | 0 s | 1.05284 | 0.52299 | 0.07597 | 5.37036 | 2.59 |
|  | 80 s | 1.22213 | 0.5428 | 0.18682 | 4.14123 | 2.48 |
|  | 150 s | 1.84248 | 0.55928 | 0.08827 | 5.02481 | 1.90 |
| 535 | 0 s | 0.93371 | 0.98321 | 0.32509 | 5.69502 | 4.13 |
|  | 80 s | 0.5886 | 1.286 | 0.49513 | 9.04721 | 7.93 |
|  | 150 s | 0.403 | 1.47416 | 0.59316 | 8.78136 | 8.03 |
| 605 | 0 s | 1.15378 | 0.6946 | 0.30225 | 4.24364 | 2.88 |
|  | 80 s | 0.66243 | 0.96688 | 0.48232 | 6.01345 | 5.10 |
|  | 150 s | 0.58607 | 1.54481 | 0.46139 | 6.35523 | 5.22 |

**Table S13.** Average fluorescence lifetime of CDs/TEOA-3@CMC under different UV irradiation times

| λ (nm) | UV light exposure time (s) | A1 (%) | τ1 (ns) | A2 (%) | τ2 (ns) | <τ> (ns) |
| --- | --- | --- | --- | --- | --- | --- |
| 445 | 0 s | 1.26753 | 0.5875 | 0.12134 | 5.42235 | 2.86 |
|  | 90 s | 1.08873 | 0.62572 | 0.17663 | 4.35577 | 2.60 |
|  | 180 s | 1.0497 | 0.57533 | 0.12438 | 4.65679 | 2.57 |
| 535 | 0 s | 0.92679 | 1.06082 | 0.27329 | 5.51318 | 3.76 |
|  | 90 s | 0.78526 | 1.16217 | 0.30785 | 8.2217 | 6.35 |
|  | 180 s | 0.72221 | 1.18615 | 0.42993 | 8.63674 | 7.24 |

**Table S14.** Average fluorescence lifetime of CDs/TEOA-4@CMC under different UV irradiation times

| λ (nm) | UV light exposure time (s) | A1 (%) | τ1 (ns) | A2 (%) | τ2 (ns) | <τ> (ns) |
| --- | --- | --- | --- | --- | --- | --- |
| 445 | 0 s | 1.14382 | 0.73321 | 0.06019 | 6.9928 | 2.82 |
|  | 120 s | 1.03479 | 0.74005 | 0.10624 | 5.23089 | 2.63 |
|  | 240 s | 1.53857 | 0.49323 | 0.10364 | 5.1913 | 2.44 |
| 535 | 0 s | 0.95122 | 1.13105 | 0.23415 | 5.2669 | 3.34 |
|  | 120 s | 0.88898 | 1.01962 | 0.34851 | 8.40887 | 6.66 |
|  | 240 s | 0.65823 | 1.2113 | 0.42804 | 9.23369 | 7.89 |

**Table S15.** Average fluorescence lifetime of CDs/TEOA-5@CMC under different UV irradiation times

| λ (nm) | UV light exposure time (s) | A1 (%) | τ1 (ns) | A2 (%) | τ2 (ns) | <τ> (ns) |
| --- | --- | --- | --- | --- | --- | --- |
| 445 | 0 s | 1.16983 | 0.78379 | 0.18784 | 4.95751 | 2.89 |
|  | 150 s | 0.82489 | 0.87878 | 0.20087 | 4.33179 | 2.76 |
|  | 300 s | 1.06128 | 0.76518 | 0.18248 | 4.51526 | 2.65 |
| 535 | 0 s | 0.74746 | 1.121 | 0.29872 | 5.13422 | 3.71 |
|  | 150 s | 0.91174 | 1.11502 | 0.30124 | 8.10639 | 6.05 |
|  | 300 s | 0.92506 | 1.21004 | 0.29763 | 8.54608 | 6.30 |

**Table S16.** Vertical excitation energy, corresponding wavelength and oscillator strength calculated from Model 1 and Model 2

| Model | Transition | Main orbital contribution | Energy (eV) | Wavelength (nm) | Oscillator strength (f) |
| --- | --- | --- | --- | --- | --- |
| 1 | S₀→S₁ | HOMO-LUMO | 2.1211 | 584.5 | 0.0334 |
| 1 | S₀→S₂ | HOMO-1-LUMO | 2.5426 | 487.6 | 0.3327 |
| 1 | S₀→S₃ | HOMO-2-LUMO | 2.9791 | 416.2 | 0.0001 |
| 1 | S₀→S₄ | HOMO-3-LUMO | 3.2145 | 385.7 | 0.0865 |
| 1 | S₀→S₅ | HOMO-4-LUMO | 3.2980 | 375.9 | 0.0005 |
| 1 | S₀→S₆ | HOMO-5-LUMO | 3.4104 | 363.6 | 0.0887 |
| 2 | S₀→S₁ | HOMO-LUMO | 2.1000 | 590.4 | 0.0347 |
| 2 | S₀→S₂ | HOMO-1-LUMO | 2.5406 | 488.0 | 0.3378 |
| 2 | S₀→S₃ | HOMO-2-LUMO | 2.9914 | 414.5 | 0.0000 |
| 2 | S₀→S₄ | HOMO-3-LUMO | 3.0540 | 406.0 | 0.0015 |
| 2 | S₀→S₅ | HOMO-4-LUMO | 3.1883 | 388.9 | 0.1010 |
| 2 | S₀→S₆ | HOMO-5-LUMO | 3.3050 | 375.2 | 0.0008 |
